# Adult-born neurons in critical period maintain hippocampal seizures via local aberrant excitatory circuits

**DOI:** 10.1038/s41392-023-01433-4

**Published:** 2023-06-07

**Authors:** Liying Chen, Yingwei Xu, Heming Cheng, Zhongxia Li, Nanxi Lai, Menghan Li, Yeping Ruan, Yang Zheng, Fan Fei, Cenglin Xu, Jiao Ma, Shuang Wang, Yan Gu, Feng Han, Zhong Chen, Yi Wang

**Affiliations:** 1 Institute of Pharmacology & Toxicology, College of Pharmaceutical Sciences, School of Medicine, Zhejiang University, Hangzhou, China; 2 Key Laboratory of Neuropharmacology and Translational Medicine of Zhejiang Province, School of Pharmaceutical Sciences, Zhejiang Chinese Medical University, Hangzhou, China; 3 Zhejiang Rehabilitation Medical Center Department, The Third Affiliated Hospital, Zhejiang Chinese Medical University, Hangzhou, China; 4 Key Laboratory of Cardiovascular & Cerebrovascular Medicine, Drug Target and Drug Discovery Center, School of Pharmacy, Nanjing Medical University, Nanjing, China

**Keywords:** Diseases of the nervous system, Neurogenesis

## Abstract

Temporal lobe epilepsy (TLE), one common type of medically refractory epilepsy, is accompanied with altered adult-born dentate granule cells (abDGCs). However, the causal role of abDGCs in recurrent seizures of TLE is not fully understood. Here, taking advantage of optogenetic and chemogenetic tools to selectively manipulate abDGCs in a reversible manner, combined with Ca^2+^ fiber photometry, trans-synaptic viral tracing, in vivo/vitro electrophysiology approaches, we aimed to test the role of abDGCs born at different period of epileptogenic insult in later recurrent seizures in mouse TLE models. We found that abDGCs were functionally inhibited during recurrent seizures. Optogenetic activation of abDGCs significantly extended, while inhibition curtailed, the seizure duration. This seizure-modulating effect was attributed to specific abDGCs born at a critical early phase after kindled status, which experienced specific type of circuit re-organization. Further, abDGCs extended seizure duration via local excitatory circuit with early-born granule cells (ebDGCs). Repeated modulation of “abDGC-ebDGC” circuit may easily induce a change of synaptic plasticity, and achieve long-term anti-seizure effects in both kindling and kainic acid-induced TLE models. Together, we demonstrate that abDGCs born at a critical period of epileptogenic insult maintain seizure duration via local aberrant excitatory circuits, and inactivation of these aberrant circuits can long-termly alleviate severity of seizures. This provides a deeper and more comprehensive understanding of the potential pathological changes of abDGCs circuit and may be helpful for the precise treatment in TLE.

## Introduction

Epilepsy is considered to be a circuit-level syndrome, which is pathologically characterized by hypersynchronous or excessive discharges with enhanced neuronal excitability. It is one of the most common neurological disorders affecting all ages with a prevalence of 0.7%. Anti-seizure medicine is the first-choice treatment for epilepsy, whereas approximately 1/3 of patients still fail to achieve seizure control, ultimately becoming drug-resistant for epilepsy.^1,2^ TLE is one of the most common types of drug resistant epilepsy in adult patients, in which epileptic seizures often initiate locally within the hippocampus and may spread secondarily throughout the entire brain.^3,4^ From a therapeutic standpoint, TLE is often medically refractory because of frequent resistance to anti-seizure medicine and surgical resection.^5,6^ Lack of control of seizures and seizure-related severe comorbidities are heavy burden on those TLE patients, families and even the society. Thus, identification of the neuronal circuit involved in seizures of TLE is necessary for the development of safe and precise interventions to treat TLE.

Although the level of adult neurogenesis is relatively low, recent data supports its critical role in physiological functions^7–9^ and various pathological CNS diseases, including epilepsy.^10,11^ Notably, epileptogenic insults such as stroke, febrile seizures, head trauma or status epilepticus (SE) may lead to transient upregulation of adult neurogenesis in the subgranular zone (SGZ)^12–14^ and later accelerate the abnormal functional integration into abDGCs,^15,16^ which are believed to have a role in later epileptogenesis. For example, Parent et al., used BrdU labeling to evaluate cell proliferation and reported a transient increase of neurogenesis after pilocarpine-elicited SE for the fist time.^12^ Further, abDGCs were found to show severe morphological abnormalities,^17,18^ locate ectopically and burst in synchrony with CA3 pyramidal cells,^15^ which have been suggested to be pro-epileptogenic. Interestingly, previous studies using pharmacological interventions to inhibit adult neurogenesis produced controversial results with regard to whether neurogenesis plays contributory or protective role in epileptogenesis.^19–21^ Recent studies using more selective genetic methods have pointed out that seizure-associated neurogenesis may directly contribute to pro-epileptogenic effects.^22–24^ Notably, in the pilocarpine-induced epilepsy model, genetic ablation of neurogenesis prior to or immediately after the induction of SE significantly reduced the frequency of spontaneous recurrent seizures (SRS),^23^ suggesting that adult neurogenesis may greatly contribute to epileptogenesis. However, selective ablation of neurogenesis may result in re-wiring of neuronal connectivity of existing neurons and abDGCs, while the direct role of abDGCs in recurrent epileptic seizures of TLE is still unknown. Recently, it has been reported that chemogenetic suppression of abDGCs generated after the pilocarpine-induced SE dramatically reduce epileptic spikes and later SRS, without interrupting neural circuit formation during epileptogenesis.^24^ This strongly suggests that abDGCs might affect not only epileptogenesis but also epileptic seizures per se, but there still remains question on how abDGCs contribute to pro-seizure neural circuits.

The features of abDGCs, including proliferation, morphology as well as functional integration, are highly heterogeneous. The electrophysiological properties and function of abDGCs varied during their maturation.^25,26^ Lybrand et al., identified that a critical period of abDGC maturation (< 2w) was closely related with the aberrant maturation of abDGCs (abnormal dendritic morphology and ectopic cell migration),^27^ which was thought to disrupt DG circuitry and cause SRS.^28,29^ Meanwhile, abDGCs generated at different stage (relative to the epileptogenic insults) were reported to be morphologically heterogeneous.^30–32^ For example, Walter et al., found that labeled DGCs which were generated 3 weeks after (newborn), 1 week before (immature), or 8 weeks before (mature) pilocarpine epileptogenesis developed heterogeneously. Specifically, almost half of labeled immature DGCs exposed to pilocarpine-epileptogenesis showed aberrant hilar basal dendrites (HBDs); compared with immature cells, newborn DGCs were even more severely affected, with 40% exhibiting HBDs and an additional 20% exhibiting migration defects; however, only 9% of mature DGCs exposed to the same insult possessed HBDs.^32^ Utilizing a rabies virus-mediated retrograde tracing strategy, recent studies mapped the upstream connectivity of hippocampal DGCs generated at different stage of seizures and revealed that they possibly differently contribute to epileptic seizures through establishment of various aberrant circuits.^24,33^ However, how they contribute to later epileptic seizures is not fully understood due to lack of specific method to map and modulate abDGCs.

Therefore, in the present study, taking advantage of optogenetic and chemogenetic tools to selectively manipulate neural circuits in a reversible manner, we revealed that abDGCs born at a critical early period bidirectionally modulate seizure duration in mouse TLE models. Combined with Ca^2+^ fiber photometry, trans-synaptic viral tracing, in vivo/vitro electrophysiology approaches, we further revealed that abDGCs experienced circuit re-organization in epilepstic brain and extended seizure duration via local excitatory circuit with early-born granule cells (ebDGCs). Repeated modulation of “abDGC-ebDGC” circuits easily induce a change of synaptic plasticity, and achieve long-term anti-seizure effects in mouse TLE models. This provides a more comprehensive understanding of the pathological alterations of abDGC network in epilepsy and may also be helpful for the development of precise therapeutics targeted at the abDGCs to spatiotemporally control TLE.

## Results

### The abDGCs are functionally inhibited during hippocampal seizure*s*

**Fig. 1:**
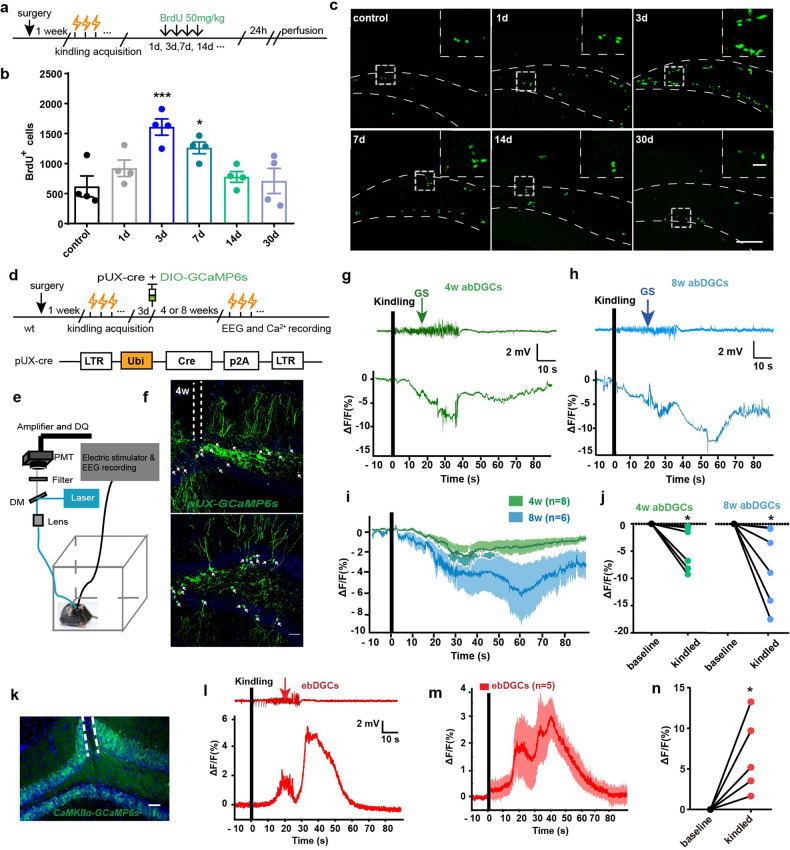
The abDGCs generated acutely after kindled status are functionally inhibited during later recurrent hippocampal seizures. **a** Experiment scheme of using BrdU to label cell proliferation in adult SGZ at different timepoints after mice being fully kindled. **b** Proliferative activity in the SGZ was significantly increased at 3 days, remained elevated at 7 days, and returned to baseline levels by 14 days after mice being fully kindled (*n* = 4 for each group *p < 0.05, ***p < 0.001, compared with control; One-way ANOVA followed by *post hoc* Dunnett test). **c** Representative images of BrdU labeling at different timepoints after mice being fully kindled (bar = 50 μm) and the enlarged images (bar = 10 μm). **d** Schematic diagram of the Ca^2+^ fiber photometry experiment. Fluorometric monitoring was carried out separately 4 or 8 weeks after the virus being injected to label 3-day abDGCs. **e** Configuration for fluorometric monitoring of Ca^2+^ signaling of abDGCs and simultaneous EEG recording during hippocampal seizures. **f** Histochemical verification of GCaMP6s expression in coronal sections in the DG (bar = 50 μm). White arrow points to the labeled abDGCs. **g**, **h** Representative GCaMP signals aligning with EEG recordings during hippocampal seizures when labeled abDGCs were 4-weeks-old (green) and 8-weeks-old (blue), respectively. The parameter of kindling stimulation is: monophasic square-wave pulses, 20 Hz, 1 ms/pulse, 40 pulses, 200μA. **i** Mean fluorescence values of abDGCs during hippocampal seizures (*n* = 8 for 4 w; *n* = 6 for 8 weeks). **j** The statistical value of ΔF/F_0_ was shown separately for each mouse in the 4-weeks-old group and 8-weeks-old group (*p < 0.05, Paired Wilcoxon-tests). **k** Histochemical verification of GCaMP6s expression in ebDGCs in coronal sections (bar = 50 μm). **l** Representative GCaMP signal of ebDGCs aligning with EEG recording during hippocampal seizure. **m** Mean fluorescence values of ebDGCs during hippocampal seizures (*n* = 5). **n** The statistical value of ΔF/F_0_ was shown for each mouse in the *CaMKII-GcaMP6s* group (*p < 0.05, Paired Wilcoxon-tests)

Firstly, we aimed to see how initial epileptic seizures affected proliferation of abDGCs, by adopting a hippocampal kindling model,^34,35^ which clinically resembles complex partial seizures with secondary generalized seizures (GS). After mice being fully kindled, we administered systemically BrdU to label mitotically active cells^12^ at different time points (Fig. 1a). Consistent with previous reports,^13^ kindling had stimulating effects on the proliferation of abDGCs in the SGZ. Quantitative analysis of BrdU labeling within the SGZ revealed a substantial increase in mitotic activity at 3 d and 7 d after kindling compared with controls and returned to baseline levels by 14 d after kindled status (Fig. 1b, c).

Then, to examine how proliferation of abDGCs would be involved in later recurrent epileptic seizures, we used Ca^2+^ fiber photometry to monitor the neural activity of abDGCs during later kindled seizures. We performed a dual-virus injection, a retroviral vector, which was previously used to target abDGCs,^25,36^ together with Cre-recombinase system to express genetically encoded Ca^2+^ indicator GCaMP6s (AAV-EF1a-DIO-GCaMP6s) in abDGCs, to “birthdate” abDGCs generated at 3 d after mice being fully kindled (named *pUX-GCaMP6s* mice, Fig. 1d). To verifiy the specificity of dual-virus strategies in labeling abDGCs with Ca^2+^ indicator GCaMP6, mice were perfused 3 weeks post virus injection and the immunostaining of doublecortin (DCX) was conducted. We found that 68.33% of GCaMP6-expressing cells were colocalized with DCX. Since the development and maturation of abDGCs is a dynamic process and DCX are only positive for a certain developmental stage of abDGCs, it is relatively difficult to use double-labeling with DCX to test the specificity, while this co-localization percentage is approximate to the published data.^37^ Thus, these results suggested that the strategies we used to label newborn abDGCs were relatively reliable (supplementary Fig. 1a–c). As the morphological and physiological phenotypes of abDGCs gradually become mature normally 4 weeks after birth,^25,38^ we measured changes in the GCaMP6s fluorescence of abDGCs by fiber photometry system with EEG monitoring, when they were 4-weeks-old and 8-weeks-old, separately (Fig. 1e, f). Aligning the GCaMP6 signals with EEG, our recordings revealed that at both 4- and 8- weeks post injection, kindled seizures coincided with decreased fluorescence signal in *pUX-GCaMP6s* mice (Fig. 1g, h). Accordingly, the mean ΔF/F_0_ recorded in abDGCs showed a significant decrease compared with the baseline state (Fig. 1i). Meanwhile, the maximum value of ΔF/F_0_ was shown separately for each trial (Fig. 1j). No significant GCaMP6s fluorescence change was observed in opsin-negative mice (supplementary Fig. 2d–f), suggesting seizure-dependent change of the neuronal activity was not due to movement artifacts. These results reveal a seizure-dependent inhibition of the neuronal activity of abDGCs, indicating both 4-weeks-old and 8-weeks-old abDGCs are directly involved in recurrent hippocampal seizures.

On the other hand, c-fos staining was also used to provide additional support for the inhibited activity of abDGCs (generated acutely after seizures) during seizures. We employed an alternative retroviral vector^24,33^ pROV-U6-ShRNA-EF1a-EGFP to “birthdate” abDGCs generated at 3 d after mice being fully kindled. After 4 weeks, mice were fully kindled again, perfusion was performed 1.5 h later and immunohistochemistry (IHC) was then conducted to evaluate c-fos expression. The results showed that only 15.3% of EGFP^+^-expressing cells were colocalized with fos (supplementary Fig. 2a–c), indicating that abDGCs generated at 3 d after mice being fully kindled may not be functionally activated during seizures. We also examined the neuronal activity of early-born dentate granule cells (ebDGCs) using Ca^2+^ fiber photometry as a comparison. In contrast, fluorescent signals recorded in ebDGCs increased during hippocampal seizures (Fig. 1k, l). Meanwhile, the mean ΔF/F_0_ also showed a significant increase and the maximum value of ΔF/F_0_ was shown separately for each trial, accordingly (Fig. 1m, n). Altogether, these results demonstrated that abDGCs and ebDGCs are functionally differently involved in recurrent seizures, and abDGCs are functionally inhibited during hippocampal seizures.

### Optogenetic activation of abDGCs extends seizure duration

**Fig. 2:**
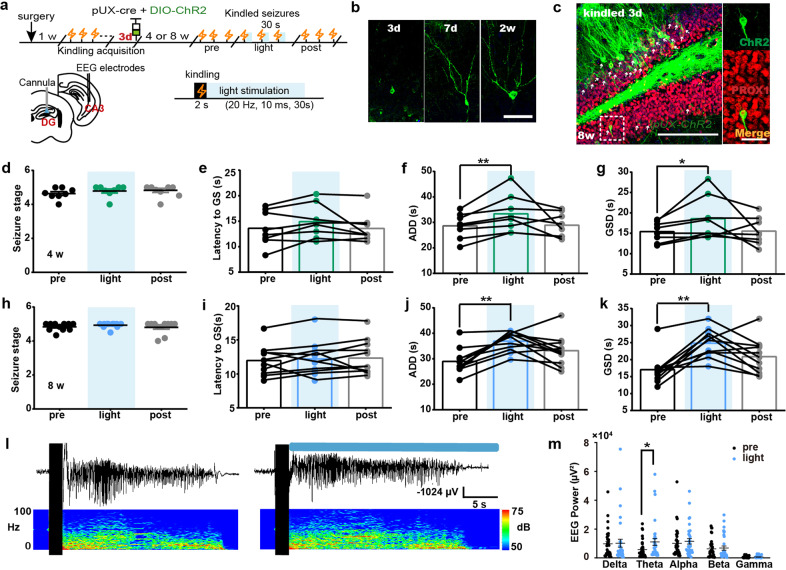
Optogenetic activation of abDGCs extends seizure duration. **a** Experiment scheme for optogenetic activation protocol in recurrent hippocampal seizures. The parameter of blue light stimulation is: 473 nm, 20 Hz, 10 ms/pulse and 600 pulses, 5 mW. **b** Representative images of EYFP-immunoreactive cells at different time points (3 days, 7 days, 2 weeks) after injection of virus cocktail (pUX-cre and AAV-EF1a-DIO-hChR2-EYFP) (bar = 50 μm). **c** Histochemical verification of ChR2-expressing abDGCs in the DG and double immunostaining of PROX1 (red) and ChR2-EYFP (green) in brain slices with 8-weeks-old abDGCs (bar = 50 μm) and the enlarged images (bar = 10 μm). White arrow points to the labeled abDGCs. **d**–**g** Effects of optogenetic activation of the 4-weeks-old abDGCs on seizure stage (**d**), latency to GS (**e**), ADD (**f**) and GSD (**g**) during hippocampal seizures (*n* = 8, *p < 0.05, **p < 0.01, Friedman-tests with post-hoc Dunn’s test for multiple comparisons). **h**–**k** Effects of optogenetic activation of the 8-weeks-old abDGCs on seizure stage (**h**), latency to GS (**i**), ADD (**j**) and GSD (**k**) during hippocampal seizures (*n* = 11, **p < 0.01, Friedman-tests with post-hoc Dunn’s test for multiple comparisons). **l** Typical EEGs and power spectrograms recorded from the hippocampus during seizures when optogenetically activating 8-weeks-old abDGCs; the solid black vertical bar indicates kindling stimulation artifact and the horizontal blue bar indicates the time for delivery of blue light. **m** Power spectral analysis of the EEGs of hippocampal seizures (*p < 0.05, Paired *t*-tests)

To further investigate the causal link between abDGCs and recurrent hippocampal seizures, we aimed to gain selective optogenetic activation of abDGCs. We microinjected AAV-EF1a-DIO-hChR2-EYFP together with pUX-cre into the DG 3 d after mice were fully kindled (named *pUX-ChR2* mice, Fig. 2a). Representative images of EYFP-immunoreactive cells at different time points (3d, 7d, 2 week) showed the morphologic development of abDGCs in *pUX-ChR2* mice (Fig. 2b), suggesting selective expression can be conferred to proliferating cells. The specificity of dual-virus strategies to selectively label abDGCs with ChR2 was also verified using IHC. The results showed that 61.97% of ChR2-expressing cells were colocalized with DCX (supplementary Fig. 3a–c). Further, IHC confirmed that ChR2 localized selectively to PROX1-expressing 8-weeks-old DGCs (82.35 ± 5.453% of EYFP^+^ neurons were PROX1^+^ neurons from 3 mice), indicating that 8-weeks-old abDGCs were mature DGCs (Fig. 2c).

To evaluate the effect of activation of abDGCs on hippocampal seizures, we applied photo-stimulation to the DG immediately after hippocampal kindling stimulation, which was similar to the closed-loop stimulation pattern. As AAV transduction has been reported to impair the generation of abGCs,^39^ here we used self-control to test seizure-modulating effect with light on-off in optogenetic experiments. A recent study by McHugh et al., characterized the spiking activity of abDGCs, reporting that the average firing rate of abDGCs was significantly higher than that of the other DG pyramidal cells (abDGCs 2.3 Hz vs DG PCs 1.0 Hz),^40^ firstly provided evidence for the natural firing frequency of abDGCs under physiological states. While we were still lacking the evidence for the firing frequency of abDGCs during seizures, we selected the frequency 20 Hz, which is significantly higher than the natural firing rate of abDGCs under physiological states, to optogenetically stimulate them, based on our experience on optogenetic activation experiments.^35,41^We found that optogenetic activation of 4-weeks-old abDGCs did not influence seizure stage, possibly due to the ceiling effect. While, activation of abDGCs significantly prolonged afterdischarge duration (ADD) and GS duration (GSD) without interfering with the latency to GS (Fig. 2d–g), suggesting abDGCs may be involved in seizure maintenance. In parallel, optogenetic activation of 8-weeks-old abDGCs also extended seizure duration by increasing ADD and GSD (Fig. 2h–k). Typical ADDs and their corresponding power spectrums were shown in Fig. 2l. Spectral composition analysis of EEGs showed that optogenetic activation significantly increased the theta band power of seizure (Fig. 2m).

Furthermore, we employed an alternative retroviral vector^24,38^ pROV-EF1a-ChR2-mCherry to “birthdate” and optogenetically activate abDGCs. IHC confirmed that ChR2 localized selectively to PROX1-expressing DGCs (91.67 ± 9.28% of mCherry^+^ 8-weeks-old neurons were PROX1^+^ neurons from 3 mice, supplementary Fig. 4a, b). We found that optogenetic activation of both 4 (supplementary Fig. 4c–f) and 8-weeks-old abDGCs in this way also increased ADD and GSD (supplementary Fig. 4g–j). However, light delivery to opsin-negative controls has no effect on hippocampal seizures (supplementary Fig. 4k–n). Altogether, these results demonstrate that selective activation of abDGCs using two distinct approaches can both aggravate seizures by increasing their duration.

### Optogenetic inhibition of the abDGCs shortens seizure duration

**Fig. 3:**
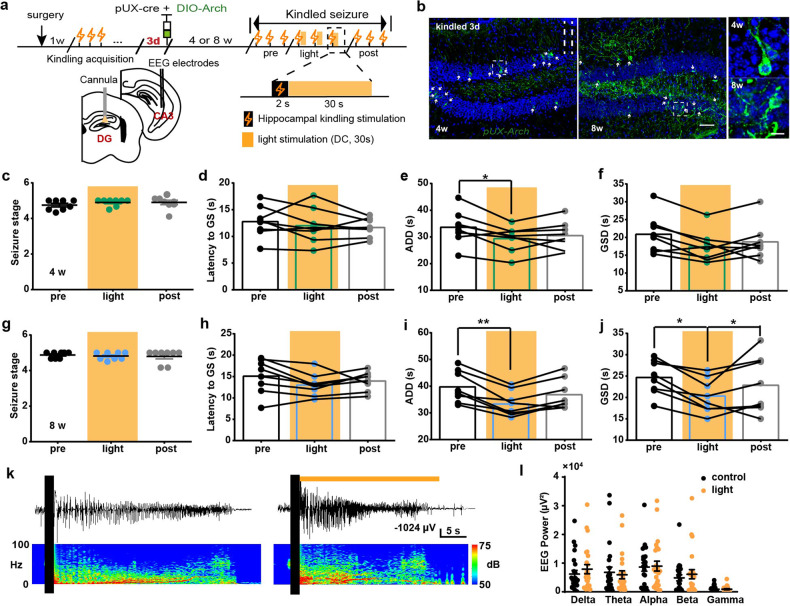
Optogenetic inhibition of abDGCs shortens seizure duration. **a** Experiment scheme for optogenetic inhibition protocol in recurrent hippocampal seizures. Continuous 30-s yellow light (589 nm, direct current, 5 mw) stimulation was applied. **b** Histochemical verification of Arch-expressing 4- and 8-weeks-old abDGCs in the DG (bar = 50 μm) and the enlarged images (bar = 10 μm). **c**–**f** Effects of optogenetic inhibition of the 4-weeks-old abDGCs on seizure stage (**c**), latency to GS (**d**), ADD (**e**) and GSD (**f**) during hippocampal seizure (*n* = 8, *p < 0.05, Friedman-tests with post-hoc Dunn’s test for multiple comparisons). **g**–**j** Effects of optogenetic inhibition of the 8-weeks-old abDGCs on seizure stage (**g**), latency to GS (**h**), ADD (**i**) and GSD (**j**) during hippocampal seizures (*n* = 8, *p < 0.05, **p < 0.01, Friedman-tests with post-hoc Dunn’s test for multiple comparisons). **k** Typical EEGs and power spectrograms recorded from the hippocampus during seizures when optogenetically inhibiting 8-weeks-old abDGCs; the solid black vertical bar indicates kindling stimulation artifact and the horizontal yellow bar indicates the time for delivery of yellow light. **l** Power spectral analysis of the EEGs of hippocampal seizures

Next, we used optogenetic method to selectively inhibit them, by injecting AAV-CAG-FLEX-ArchT-EGFP with pUX-cre into the DG (named *pUX-Arch* mice, Fig. 3a, b). To test the specificity of the retrovirus we used in the experiments, DCX expression was evaluated in *pUX-Arch* mice and we found that ~66.3% of retrovirus labeled cells were DCX^+^ (supplementary Fig. 5a, b). As the “natural” inhibition of abDGCs during seizures evolved gradually with the seizure development, optogenetics started immediately after the kindling stimulation would produce quicker and more powerful inhibition on abDGCs. We found that optogenetic inhibition of 4- and 8-weeks-old abDGCs, by the application of 589-nm yellow light, mitigated the severity of kindled seizures via shortening the ADD and GSD in *pUX-Arch* mice (Fig. 3c–j). Meanwhile, the seizure stage and the latency to GS were still left unaffected, suggesting abDGCs were involved in the maintenance of hippocampal seizures. Typical ADDs and their corresponding power spectrums were shown in Fig. 3k. Spectral composition analysis of EEGs showed that optogenetic inhibition of abDGCs did not affect seizure intensity in various rhythm bands (Fig. 3l). Here, interestingly, we found that optogenetic activation of abDGCs increased theta power, while inhibition did not change it, suggesting theta power in EEG of hippocampal seizure may not be pathological, but rather physiological and revelant to other functions. Thus, the above results indicate that the abDGCs generated at 3 days after mice are fully kindled bi-directionally modulate maintenance of hippocampal seizures.

### The abDGCs born at other stages do not affect hippocampal seizures

**Fig. 4:**
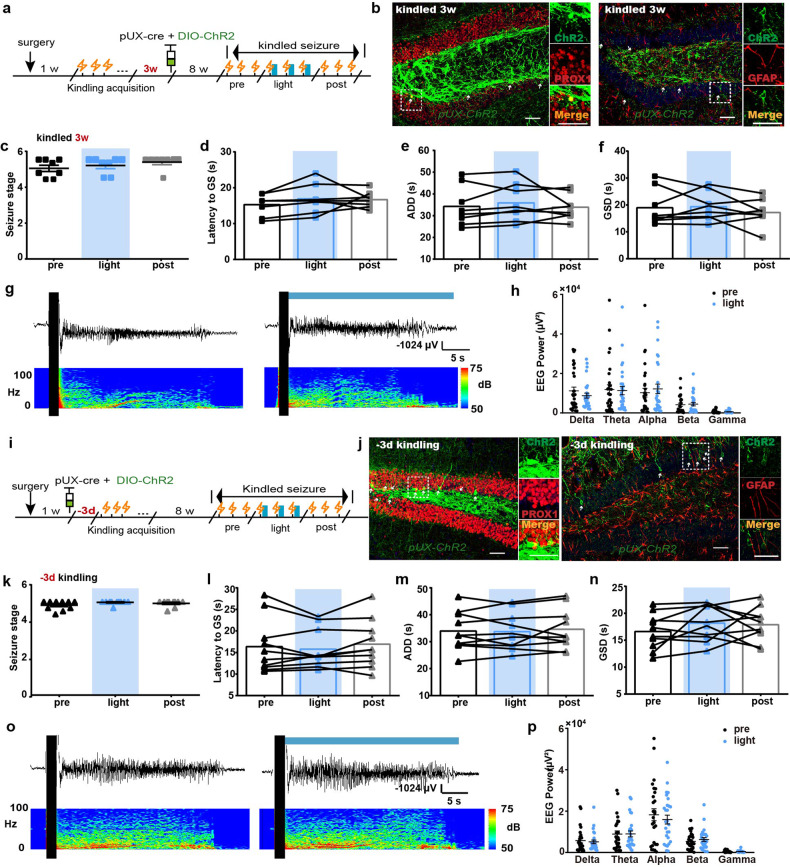
The abDGCs born at other stages of kindled status do not affect later recurrent hippocampal seizures. **a** Experiment scheme for optogenetic activation of abDGCs born at 3w after mice being fully kindled. **b** Histochemical verification of ChR2-expressing 8-weeks-old abDGCs in the DG. Left, double immunostaining of ChR2-EYFP (green) and PROX1 (red) and the enlarged images (bar = 50 μm). Right, double immunostaining of ChR2-EYFP (green) and GFAP (red) and the enlarged images (bar = 50 μm). **c–f** Effects of optogenetic activation of 8-weeks-old abDGCs, born 3 weeks after mice being fully kindled, on seizure stage (**c**), latency to GS (**d**), ADD (**e**) and GSD (**f**) during hippocampal seizures (*n* = 8). **g** Typical EEGs and power spectrograms recorded at the hippocampus during seizures when optogenetically activating 8-weeks-old abDGCs born at 3w after kindled status; the solid black vertical bar indicates kindling stimulation artifact and the solid horizontal blue bar indicates the time for blue light. **h** Power spectral analysis of the EEGs of hippocampal seizures. **i** Experiment scheme for optogenetic activation of abDGCs generated at −3d before kindling acquisition. **j** Histochemical verification of ChR2-expressing 8-weeks-old abDGCs in the DG. Left, double immunostaining of ChR2-EYFP (green) and PROX1 (red) and the enlarged images (bar = 50 μm). Right, double immunostaining of ChR2-EYFP (green) and GFAP (red) and the enlarged images (bar = 50 μm). **k–n** Effects of optogenetic activation of 8-weeks-old abDGCs, born 3 days before kindling acquisition, on seizure stage (**k**), latency to GS (**l**), ADD (**m**) and GSD (**n**) during hippocampal seizures (*n* = 10). **o** Typical EEGs and power spectrograms recorded at the hippocampus during seizures when optogenetically activating 8-weeks-old abDGCs born at −3d before kindling; the solid black vertical bar indicates kindling stimulation artifact and the solid horizontal blue bar indicates the time for blue light. **p** Power spectral analysis of the EEGs of hippocampal seizures

Next, we used optogenetic tools to dissect the sole contribution of abDGCs born at different stages in hippocampal seizures. In contrast to the initial transitory proliferative surge in the SGZ after kindled status, hippocampal neurogenesis, however, returns to the baseline level after 2 weeks.^42^ Here, instead of 3 days, a viral cocktail of pUX-cre and AAV-EF1a-DIO-hChR2-eYFP were microinjected 3 weeks after mice being fully kindled (Fig. 4a). IHC experiments confirmed that ChR2 localized selectively to PROX1-expressing 8-weeks-old abDGCs (74.33 ± 7.435% of 8-weeks-old EYFP^+^ neurons were PROX1^+^ neurons), while none was GFAP^+^ cell, indicating that most abDGCs born in the chronic phase after kindled status also mature into DGCs instead of astrocytes (Fig. 4b). We found that optogenetic activation of 8-weeks-old abDGCs exerted no significant effect on ADD, GSD, seizure stage and the latency to GS (Fig. 4c–f). Typical ADDs and their corresponding power spectrums were shown in Fig. 4g. Spectral composition analysis of EEGs showed no significant change of each band power (Fig. 4h).

Further, immature DGCs born before the epileptogenic insult might also integrate improperly into the DG during epileptogenesis.^14,43^ Thus, the viral cocktail was also given 3 days before the beginning of kindling, approximately 1 week before mice being fully kindled (Fig. 4i). IHC also confirmed that ChR2 localized selectively to PROX1-expressing 8-weeks-old abDGCs but not GFAP^+^ glia (71.33 ± 4.677% of 8-weeks-old EYFP^+^ neurons were PROX1^+^ neurons, Fig. 4j). Unexpectedly, we found that optogenetic activation of 8-weeks-old abDGCs exerted no significant effect on ADD, GSD, seizure stage and the latency to GS (Fig. 4k–n). Typical ADDs and their corresponding power spectrums were shown in Fig. 4o. Spectral composition analysis of EEGs showed no significant change of each band power (Fig. 4p). Similarly, optogenetic inhibition of abDGCs born at 3 d before kindling also exerted no significant effect on seizure stage, latency to GS, ADD, and GSD (supplementary Fig. 6a–d). In addition, we also conducted fiber photometry experiments to assess the functional activity of 8-week-old abDGCs born at 3 weeks after kindled status. We found that although optogenetic activation of abDGCs born at 3 weeks after kindled status failed to intervene hippocampal seizures, they were functionally significantly activated during seizures (supplementary Fig. 6e–h). On the other hand, we calculated the number of total labeled abDGCs born at different timepoints and found that abDGCs born at 3 d after fully kindled were significantly more than 3 weeks after, while there is no significant correlation between number of total labeled neurons and their seizure-modulating effects (supplementary Fig. 7a–c). However, after calculating the number of ectopic abDGCs (located ectopically in the hilus), we found that seizure-modulating effect was proportional to the number of ectopic abDGCs (supplementary Fig. 7d), which is consistent with the previous study by Zhou et al.^24^ This suggests that number of ectopic abDGCs may account for the different effect of abDGCs born at different timepoints.

Additionally, we used a dual-virus tracing method (combining rabies virus-mediated retrograde trans-synaptic tracing with retroviral birthdating) to identify and compare the presynaptic inputs onto abDGCs generated at different timepoints relative to seizures. Generally, seizures influenced the connectivity ratio universally, which was defined as the number of input neurons per starter cell; meanwhile, such changes usually depended on the relative age of DGCs with regard to the seizure onsets. We found that abDGCs born acutely (3 days) after fully kindled showed significantly increased connectivity ratio; specifically, they received augmented inputs from neurons located both in the hippocampus and entorhinal cortex (EC). However, ebDGCs and abDGCs generated 3 weeks after fully kindled showed no significant changes in connectivity ratio from any of these areas (supplementary Fig. 8). According to the previous report,^33^ hippocampus DGCs receive axonal innervation diversely from many different brain structures, most of which convey excitatory signals. Notably, less attention has been paid to the GABAergic inputs of DGCs. However, as we have demonstrated in Fig. 1 and supplementary Fig. 6 using fluorometric monitoring of Ca^2+^ signaling; interestingly, while abDGCs born at 3 days after kindling were functionally inhibited, ebDGCs and abDGCs born 3 weeks after fully kindled were activated. Thus, distinguishing the inhibitory inputs of DGCs generated at different timepoints relative to seizures may also be of great importance. Calculating the input cells co-localized with GABA, we found that abDGCs generated acutely after seizures showed significantly increased percentage of GABA positive input cells in various subregions of hippocampus (supplementary Fig. 9), which may accout for the functional inhibition of abDGC born acutly after seizures. These above results indicated that abDGCs born at different timepoints experience different types of circuit re-organization.

Collectively, these data demonstrate that there is a critical early time period (3 days after fully kindled) after epileptogenic insult, for abDGCs to be involved in hippocampal seizures.

### The abDGCs extend seizure duration via local recurrent excitatory circuit with ebDGCs

**Fig. 5:**
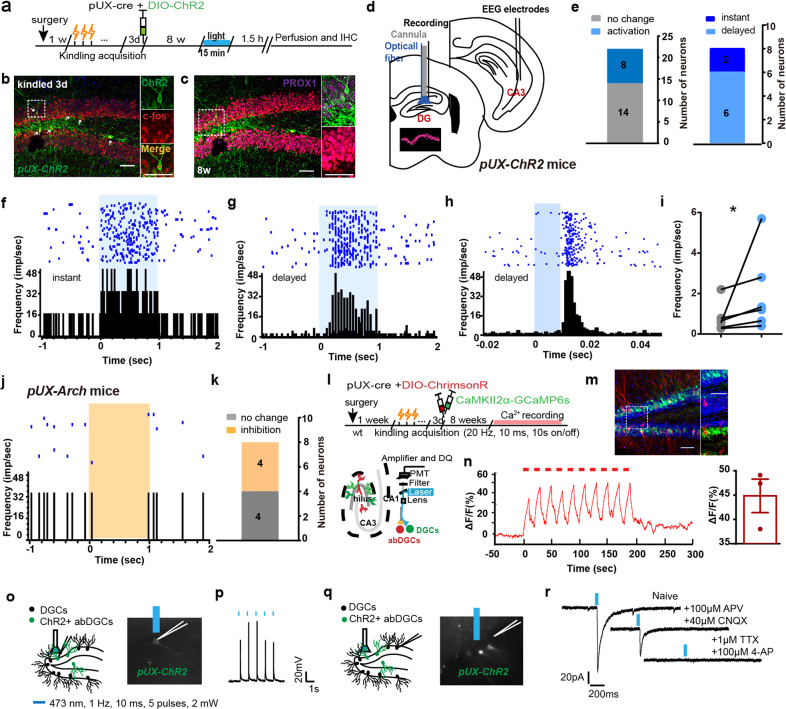
The abDGCs activate local ebDGCs in epileptic mice. **a** Experimental scheme for optogenetic activation of 8-weeks-old abDGCs born at 3 days after kindled status leads to c-fos expression in both ChR2-expressing abDGCs and other PROX1^+^ neurons in the GCL. **b** Immunostaining of c-fos (red) and ChR2-EYFP (green) and the enlarged images (bar = 50 μm); **c** Immunostaining of PROX1 (purple), ChR2-EYFP (green) and c-fos (red) and the enlarged images (bar = 50 μm). **d** Scheme of experiment for in vivo single unit recording when optogenetically activating 8-weeks-old abDGCs born at 3d after kindled status. Insert, the typical DGC spike waveform. **e** Statistics of firing response of recorded DGCs with photo-stimulation of ChR2-expressing abDGCs in kindled status. **f**, **g** Representative peri-event raster histogram of DGCs firing in response to photo-stimulation of ChR2-expressing abDGCs (10 ms bins) with either instant (**f**) or delayed (**g**) latency. **h** Peristimulus time histogram of the representative DGC (aligned by the pulse light onset, blue rectangle) reveals its response frequency to the photo-stimulation (peak response latency ~15 ms). **i** The statistical value of firing rate was shown separately for each neuron (*n* = 6) (*p < 0.05, Paired Wilcoxon-tests). **j** Representative peri-event raster histogram of DGC firing in response to photo-stimulation of Arch-expressing abDGCs (10 ms bins). **k** Statistics of firing response of recorded DGCs with photo-inhibition of ArR2-expressing abDGCs in kindled status. **l** Experiment scheme for the Ca^2+^ fiber photometry experiment during optogenetic activation of abDGCs. **m** Immunostaining of ChrimsonR (red) and GCaMP6s (green) expression in the DG and the enlarged images (bar = 50 μm). **n** Left panel: Representative trace showed that optogenetic activation of abDGCs (10 s on-off, 635 nm) increased Ca^2+^ level in the mature ebDGCs reliably. Right panel: The statistical value of ΔF/F_0_ was shown separately for each mouse, which was calculated by averaging the peak ΔF/F_0_ values (*n* = 3). **o** Scheme of experiment for in vitro electrophysiology in the acute brain slices containing DG of kindled mice, photo-stimulation of ChR2-epressing abDGCs and whole-cell recording of them. **p** Typical whole-cell current recordings from ChR2-positive abDGCs. Membrane potential responses to a train of 10 ms pulses of blue light at 1 Hz. **q** Scheme of experiment for in vitro electrophysiology in the acute brain slices containing DG of kindling mice, photo-stimulation of ChR2-epressing abDGCs and whole-cell recording of neighboring ebDGCs. **r** Light-evoked excitatory postsynaptic currents (EPSCs) were recorded in ebDGCs during photo-stimulation (473 nm, 1 Hz, 10 ms, 5 pulses, 2 mW) of ChR2-expressing abDGCs in DG in normal ACSF (naive) in the presence of tetrodotoxin (TTX, 1 μM) and 4-amynopyridine (4-AP, 100 μM), and in the presence of the glutamate receptor antagonist D-2-Amino-5-phosphonovaleric acid (APV, 100 μM) and 6-cyano-7-nitroquinoxaline-2,3-dione. (CNQX, 40 μM)

Next, we aimed to see how the abDGCs born within above mentioned critical time period form pro-seizure neural circuits. We found that optogenetic activation of 8-weeks-old abDGCs born at 3 d after kindled status alone (without kindling stimulation) led to a large amount of c-fos expression in both ChR2-expressing abDGCs themselves and other neurons in the granule cell layer (GCL) (Fig. 5a, b). On the contrary, optogenetic activation of abDGCs in non-kindled (naïve) mice leads to sparse c-fos expression in GCL, with only several abDGCs located right under the optic fiber were c-fos^+^ (supplementary Fig. 10a, b), further suggesting circuit rearrangement of abDGC in epilepsy. Further, IHC verified that c-fos^+^ neurons were highly co-localized with PROX1-expressing neurons (94.73 ± 2.106% of c-fos^+^ neurons were PROX1^+^ neurons)(Fig. 5c), indicating that ChR2-expressing abDGCs can be optogenetically activated by the blue light and abDGCs may form excitatory connectivity with neighboring ebDGCs.

Furthermore, we employed in vivo extracellular recordings and photo-stimulation to confirm the functional connection between ChR2-expressing abDGCs and ebDGCs in anesthetized *pUX-ChR2* mice (Fig. 5d). According to previously published criteria,^44,45^ we discriminated DGCs based on their waveform and autocorrelogram of firing characteristics. The recorded DGCs (8 of 22) were found to be increased in spiking rates with different latencies by photostimulation. Specially, 6 out of 8 DGCs were found to be increased in spiking rates with an approximate 15-ms of latency (Fig. 5e–h). As the firing rate of ChR2-expressing neurons were usually immediately activated within much shorter latency (<4 ms) by the light stimulation,^46,47^ our data suggested ebDGCs might be activated by the pulsed laser from the upstream terminals of abDGCs. Conversely, optogenetic inhibition of Arch-expressing abDGCs were found to decrease the firing rate of DGCs in anesthetized *pUX-Arch* mice (Fig. 5i, j). On the other hand, we injected pUX-cre and AAV-hSyn-DIO-ChrimsonR-mCherry cocktail virus to label abDGCs. The ChrimsonR-expressed abDGCs can be functionally activated by the red light. Meanwhile, we also injected AAV-CaMKIIα-GCaMP6s-EGFP to label already mature ebDGC (Fig. 5l, m). We found that optogenetic activation of abDGCs (10 s on-off) largely increased Ca^2+^ level in the mature ebDGCs reliably (Fig. 5n), indicating that excitatory connections between abDGC and ebDGC.

Furthermore, we performed in vitro electrophysiology to confirm the functional connection between abDGCs and ebDGCs. Firstly, to test whether ChR2-labeled abDGCs can be reliably photo-activated, we conducted whole-cell current-clamp recordings of ChR2^+^ abDGCs in acute DG slices of kindled mice. Flashing blue light pulses (473 nm) were delivered to the slice and ChR2^+^ abDGCs recorded fired single action potentials in response to each light pulse (Fig. 5o, p). Then, glutamate-mediated synaptic currents were evoked by flashing blue light pulses during whole-cell recording from ebDGCs. Tetrodotoxin (TTX) and 4-amynopyridine (4-AP) are often used to block action potential-dependent synaptic transmission in indirect circuits; thus, the light-evoked excitatory postsynaptic currents (EPSCs) still existed in the presence of them in our experiments, demonstrating the existence of direct monosynaptic input from abDGCs to ebDGCs. The synaptic currents were eliminated when the glutaminergic receptor antagonists D-APV and CNQX were applied (Fig. 5q, r), confirming the transmission of glutamate. Altogether, these results indicate that activation of abDGCs can result in increased neuronal activity of neighboring ebDGCs in the DG.

**Fig. 6:**
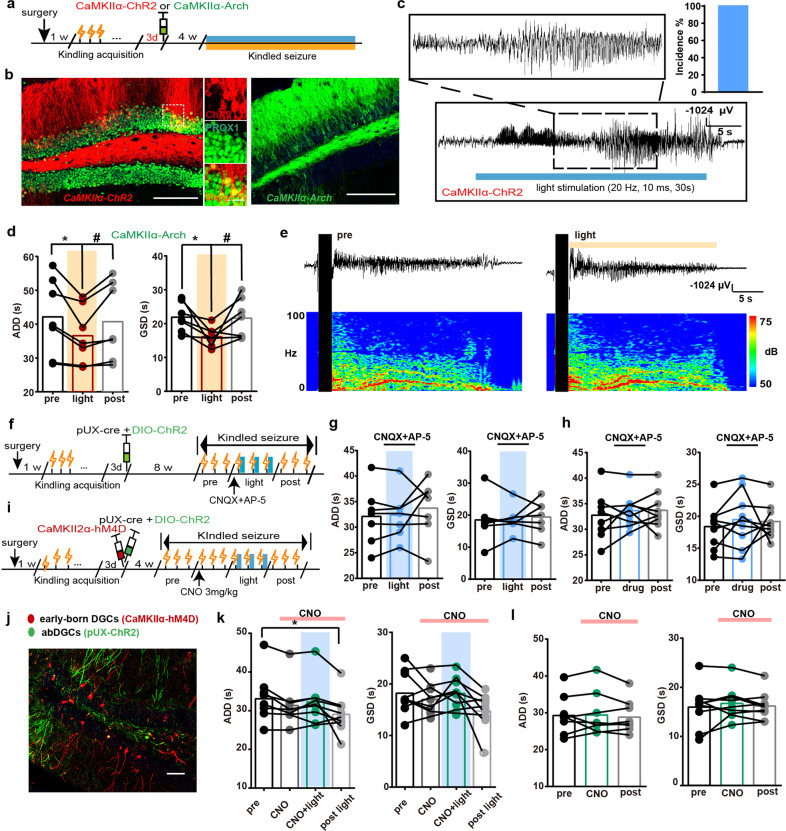
The abDGCs extend seizure duration via local recurrent excitatory circuit with ebDGCs. **a** Experiment scheme for optogenetic activation/inhibition of ebDGCs. **b** Histochemical verification of ChR2- and Arch-expressing ebDGCs in the GCL. Left, immunostaining of ChR2-mCherry (red) and PROX1 (green) (bar = 50 μm) and the enlarged images of co-localization of both stains (bar = 10 μm); Right, histochemical verification of ArchT-expressing ebDGCs in the DG (bar = 50 μm). **c** Light delivery to activate ebDGCs was sufficient to induce seizures in kindled *CaMKIIα-ChR2* mice. Left panel: Typical EEGs of ebDGC activation-evoked seizure. Right panel: The incidence of ebDGC activation-induced seizures was 100% (*n* = 4). **d** Inhibition of ebDGCs shortened the ADD and GSD during hippocampal seizures in *CaMKIIα-Arch* mice (*n* = 7, *p < 0.05, **p < 0.01, Friedman-tests with post-hoc Dunn’s test for multiple comparisons). **e** Typical EEGs and power spectrograms recorded from hippocampus when optogenetically inhibiting ebDGCs; the solid black vertical bar indicates kindling stimulation artifact and the horizontal yellow bar indicates the time for yellow light. **f** Experiment scheme for optogenetic activation protocol in the presence of glutamate antagonists (intra-DG injection, CNQX 10 μM plus AP-5 25 μM, 1:1, 0.5 μL). The drugs were injected 5 min before the insertion of optic fiber to deliver blue light. **g** Optogenetic activation of abDGCs in *pUX-ChR2* did not have any pro-seizure effects in the presence of intra-DG injection of glutamate antagonists (*n* = 7). **h** Intra-DG injection of glutamate blocker cocktails alone did not influence the ADD and GSD. **i** Experiment scheme for chemogenetic silencing of ebDGC and simultaneous optogenetic activation of abDGCs. CNO was injected (3.0 mg/kg, i.p.) 30 min before the 4th stimulation. **j** Representative histochemical image of ChR2-EYFP-expressing abDGCs (green) and hM4D-mCherry-expressing ebDGCs (red)（bar = 50 µm). **k** Effects of optogenetic activation of abDGCs on kindled seizures, while simultaneously chemogenetic inhibiting ebDGCs (*n* = 9, *p < 0.05, Friedman-tests with post-hoc Dunn’s test for multiple comparisons). Mice using in this experiment were tested beforehand to ensure that optogenetic activating of abDGCs reliably prolonged seizure duration. **l** CNO treatment alone did not influence the ADD and GSD of control animals injected with AAV-CamKIIα-GFP (*n* = 9)

Then, we aimed to evaluate whether ebDGCs in the GCL are involved in hippocampal seizures. Three days after the mice were fully kindled, AAV-CaMKIIα-ChR2-mCherry and AAV-CaMKIIα-Arch-EYFP were used to label and modulate ebDGCs as previous studies (named *CaMKIIα-ChR2* or *CaMKIIα-Arch* mice respectively, Fig. 6a).^48,49^ IHC confirmed that ChR2 was successfully expressed on DGCs (99.13% ± 5.332 of mCherry^+^ neurons were PROX1^+^ neurons) (Fig. 6b). We found that directly optogenetic activation of ebDGCs was capable of inducing seizures in kindled mice without kindling stimulation in *CaMKIIα-ChR2* mice (Fig. 6c), while optogenetic inhibition of ebDGCs decreased the ADD and GSD in *CaMKIIα-Arch* mice (Fig. 6d). However, different from the photo-stimulation of abDGCs, the severity of seizure (ADD and GSD) returned to their baseline level after withdrawal of the light. Typical ADDs and their corresponding power spectrums were also shown (Fig. 6e). These data suggest that ebDGCs in the GCL also directly contribute to hippocampal seizures.

Further, to examine whether the local ebDGCs were essential for mediating the pro-seizure effect of abDGCs, we used a pharmacological method to block excitatory glutamatergic transmission in the DG. We found that optogenetic activation of abDGCs did not have any pro-seizure effects in the presence of intra-DG injection of cocktail blockades of glutamate receptors CNQX and AP-5 (Fig. 6f, g), suggesting local glutamatergic transmission was required for pro-seizure effects of abDGCs. Surprisingly, the injection of cocktail blockades alone did not affect hippocampal seizure (Fig. 6h), which may be due to the possibility that glutamatergic transmission was inhibited in both glutamatergic and GABAergic neurons. Meanwhile, to more selectively silence ebDGCs, we focally injected chemogenetic virus AAV-CaMKIIα-hM4Di-mCherry in *pUX-ChR2* mice, thus inactivation of ebDGCs can be achieved simultaneously when photo-activating ChR2-expressing abDGCs (Fig. 6i, j). The *pUX-ChR2* mice used in this experiment were given blue light stimulations beforehand to confirm that optogenetic activating of abDGCs reliably prolonged seizure duration. We found that chemogenetic inhibition of ebDGCs alone significantly truncated ADD compared with control; Meanwhile, optogenetic activation of abDGCs cannot reproduce its pro-seizure effect upon the treatment of Clozapine N-oxide (CNO) (Fig. 6k). Moreover, we did not observe any significant reduction in seizure severity during CNO treatment in control mice (Fig. 6l), indicating that the anti-seizure effect was not due to the drug effect of CNO itself. In summary, all the above results indicate that ebDGCs may be the downstream effector mediating the pro-seizure effect of abDGCs. The abDGCs generated within a critical early period extended seizure duration by local recurrent excitatory circuits with ebDGCs.

### Repeated inhibition of abDGCs long-termly alleviates hippocampal seizures in TLE models

**Fig. 7:**
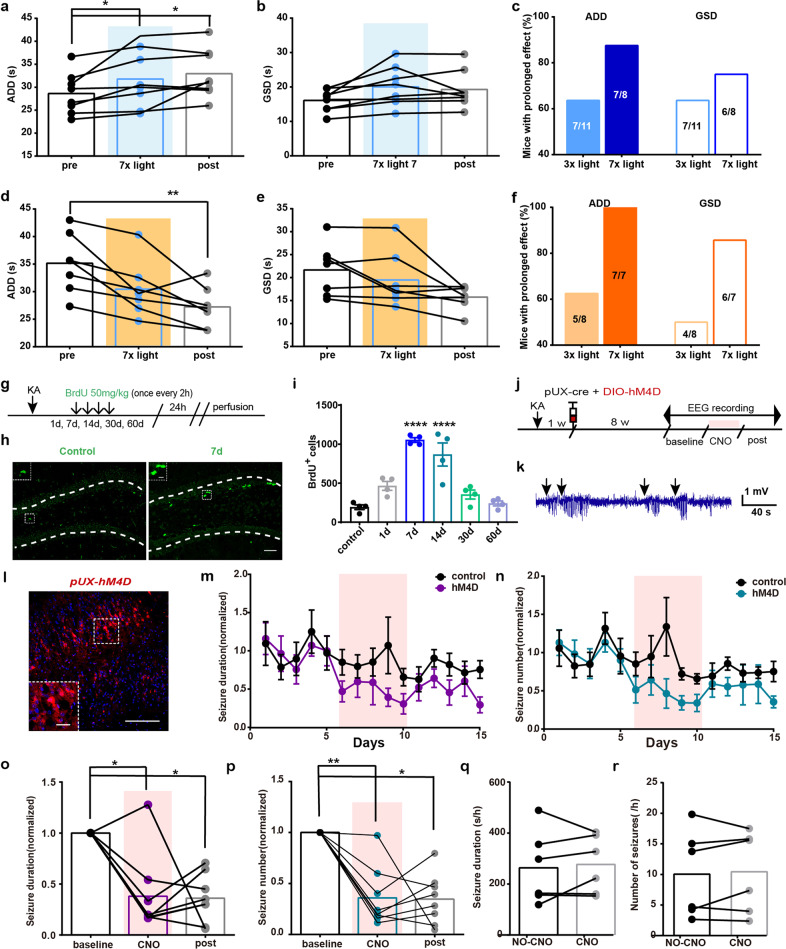
Repeated inhibition of abDGCs long-termly alleviates hippocampal seizures in TLE models. **a**, **b** Effects of 7-times repeated photo-activation of the 8-weeks-old abDGCs on ADD (**a**) and GSD (**b**) during hippocampal seizures (*n* = 8, *p < 0.05, Friedman-tests with post-hoc Dunn’s test for multiple comparisons). **c** Statistics of percentage of mice maintained their increased ADD (post ADD > pre ADD) or GSD (post GSD > pre GSD) after receiving 3- or 7-times repeated photo-activations. **d**, **e** Effects of 7-times repeated photo-inhibition of the 8-weeks-old abDGCs on ADD (**d**) and GSD (**e**) during hippocampal seizures (*n* = 7, **p < 0.01, Friedman-tests with post-hoc Dunn’s test for multiple comparisons). **f** Statistics of percentage of mice maintained their decreased ADD (post ADD < pre ADD) or GSD (post GSD < pre GSD) after receiving 3- or 7-times repeated photo-inhibitions. **g** Experiment scheme of the cell proliferation in SGZ in KA chronic epilepsy model. **h**, **i** Proliferative activity in the SGZ was significantly increased at 7 d, remained elevated at 14 d, and returned to baseline levels by 30 d after SE (*n* = 4 for each group; ****p < 0.0001, compared with control; One-way ANOVA followed by *post hoc* Dunnett test). Proliferative activity was represented by BrdU-immunostaining in the SGZ ipsilateral to the electrode-implanted hippocampi (bar = 50 μm) and the enlarged images (bar = 10 μm). **j** Experiment scheme of virus delivery and chemogenetic inhibition protocol in KA chronic epilepsy model. **k** Typical EEGs of SRS in chronic KA epilepsy model. The black arrows indicate the beginning of seizure events. **l** Histochemical verification of hM4D-mCherry-expressing abDGCs in the DG (bar = 50 μm) and the enlarged images (bar = 10 μm). **m**, **n** Effects of chemogenetic inhibition of 8-weeks-old abDGCs on seizure duration (**m**) and frequency (**n**) each day in *pUX-hM4D* mice (*n* = 8 for hM4D group, *n* = 6 for control group). **o**, **p** The statistical summary of the effects of chemogenetic inhibition of 8-weeks-old abDGCs on seizure duration (total seizure duration/8 h) (**o**) and seizure frequency (total number of seizures/8 h) (**p**) in *pUX-hM4D* mice (*n* = 8, *p < 0.05, **p < 0.01, Friedman-tests with post-hoc Dunn’s test for multiple comparisons). **q**, **r** Effects of CNO alone on seizure duration (**q**) and seizure frequency (**r**) in chronic control animals (*n* = 6)

Although activation of abDGCs significantly increased, while inhibition decreased, duration of hippocampal seizure (during light), seizure intensity after the withdrawal of photo-stimulation diverged: for some mice, the ADD or GSD returned to their initial state (pre), whereas for others the ADD or GSD maintained their increased or decreased tendency, suggesting that there may be a change of synaptic plasticity after modulation of abDGCs. To further investigate the maintenance of performance post photo-stimulation, animals were given long-term 7-times repeated photo-stimulations instead of 3-times. Increased percentage of mice were found to maintain their increased or decreased ADD and GSD post photo-stimulation after receiving 7-times photo-stimulations (Fig. 7a–f). This long-term effect without further photo-stimulation suggests that modulation of abDGCs may lead to long-term change in synaptic plasticity.

Furthermore, to investigate the role of abDGCs generated after SE in chronic TLE, we used a well-established model of TLE with KA injected unilaterally into the right dorsal hippocampus.^34,35^ In this model, we verified upregulation of BrdU^+^ cell proliferation in the DG after SE (Fig. 7g–i).^50^ We utilized retroviral delivery of synthetic G-coupled receptor hM4Dq to inactivate DGCs born 7d after SE (named *pUX-hM4D* mice). To verify the specificity of dual-virus strategies in labeling abDGCs with hM4Dq, mice were perfused 3 weeks post virus injection and the immunostaining of DCX was conducted. We found that 68.3% of hM4Dq-expressing cells were colocalized with DCX, suggesting that the strategies we used to label newborn abDGCs were relatively reliable (Supplementary Fig. 11a–c). Then, we assessed the functional impact of long-term chemogenetic inactivation of 8-weeks-old abDGCs on SRS using continuous EEG monitoring for 15 days (Fig. 7j–l). The 5-day consistent treatment of CNO reduced total seizure duration and number of SRS in chronic epileptic *pUX-hM4D* mice during 8 h-observation period each day (Fig. 7m–p); meanwhile, CNO exerted no significant effect on control animals (Fig. 7q, r). In accordance with the change of synaptic plasticity resulted from the optogenetic modulation of abDGCs as we described above, the protective effect of chemogenetic silencing of abDGCs was also long-term and didn’t return to baseline level after CNO treatment ended. These data demonstrate that repeated inhibition of abDGCs easily produce long-term anti-seizure effect in both kindling- and KA-induced TLE models.

## Discussion

Initial epileptogenic insults result in aberrant hippocampal neurogenesis, which includes increased proliferation of neural stem cells/neural progenitors and morphological abnormalities of abDGCs,^12,13,29,43^ which is believed to play contributory role in epileptogenesis.^19–23^ However, the nature of abDGCs in onset of epileptic seizure is largely unknown because of lack of specific method to map and modulate abDGCs. Here, taking advantage of optogenetics and chemogenetics, we selectively and reversibly manipulate abDGCs and demonstrate an essential role for them in the maintenance of hippocampal seizures in TLE models. Notably, fiber photometry of Ca^2+^ signaling showed that abDGCs born at 3 d after mice being fully kindled (early phase) were functionally inhibited during hippocampal seizures. Selective activation or inhibition of them significantly increased or decreased seizure duration in hippocampal kindling model, indicating that abDGCs exert their influence mainly by prolonging or curtailing seizure duration instead of affecting the initiation of seizure. On the other hand, in chronic KA model, specific suppression of abDGCs born at early phase after SE dramatically reduced the duration and frequency of SRS, suggesting that the abDGCs generated in the early phase of epileptogenic insults was critical as a potential therapeutic target for later controlling of seizures. The pro-seizure effect of abDGCs was also recently confirmed in pilocarpine-induced SRS model,^23,24^ indicating that the role of abDGCs is universal across different epilepsy models. Interestingly, we found that the abDGCs born at other stages, including 1 week before and 3 weeks after kindled status, is not effective in seizure control. It is possible that distinct populations of abDGCs born at different stage may make different and perhaps even opposing contributions to epileptic seizures, which may also help to explain the long-lasting controversies regarding the function of abDGCs. Indeed, apart from evidence of morphological heterogeneity,^31,32^ there was also report partly supporting the functional heterogeneity of abDGCs generated at different stage relative to SE.^23^ As was demonstrated by Cho and colleagues, although the single round of ganciclovir (GCV) treatment prior to acute SE resulted in a significant reduction of SRS; surprisingly, they observed no significant effect after relatively more complete ablation of neurogenesis, which was achieved by two rounds of GCV injection (pre plus post-ablation). These results are in consistent with the hypothesis that populations of abDGCs generated before and after the epileptogenic insult contain a mix of pathological and protective neurons. In that way, the net effect of abDGCs may depend on a counterbalance between heterogeneous abDGCs born at different stages. Together, our work suggests abDGCs may be used as a promising target for controlling of epileptic seizures; however, the time for treatment should be carefully selected. For patients with epileptogenic brain injury, the abDGCs generated especially at early stage during the latent period before the establishment of epilepsy should be attached to great importance.

In addition, the controversial behavioral impact of adult neurogenesis according to previous studies may be attributed to the intervention of the mixture of young abDGCs with increased plasticity and mature abDGCs. Previous studies showed that ablation of young abDGCs in physiological status led to a larger spread of excitatory activity in the DG since GABAergic cells might be the major postsynaptic targets of them.^51–54^ Furthermore, Luna et al., found that under normal conditions, young abDGCs directly inhibit or excite mature DGCs in response to inputs from lateral and medial EC separately through different glutamatergic receptors; however, mature DGCs don’t share these mechanisms, which is consistent with the fact that under normal conditions mature DGCs do not form direct connections with each other.^55^ In our study, we found that abDGCs generated within the critical period exert their pro-seizure effects when they matured and how they influenced local network properties in the DG was also investigated. The results of in vivo electrophysiology, Ca^2+^ fiber photometry and c-fos IHC showed that abDGCs generated acutely after seizures (when they matured) can activate the neighboring ebDGCs; the results of in vitro electrophysiology further demonstrated the direct monosynaptic glutamatergic input from abDGCs to ebDGCs. In addition, pharmacological approaches, which non-specifically blocked the function of local glutamate receptors, as well as chemogenetic silencing of ebDGCs reversed the pro-epileptic effect of optogenetic activation of mature abDGCs; meanwhile, inhibition of ebDGCs were able to mimic the anti-seizure effects of inactivation of abDGCs. Our results suggest that abDGCs generated acutely after kindled status form excitatory synapses onto neighboring ebDGCs and lengthen seizures mainly through activating downstream ebDGCs. Substantial excitatory input to mature DGCs is supposed to bring a large amount of excitatory drive, however, they are actually sparsely activated owing to feedforward and feedback inhibition, which supports the “gate” theory of DG.^40,56,57^ In epileptic condition, however, aberrantly developed and abnormally integrated abDGCs contributed to hippocampal excitability and functional disruption of the dentate gate when they matured.^24,33^ Our results indicated the formation of abnormal excitatory recurrent circuits in the DG after epileptogenic insults which may underlie the production and amplification of excessive excitatory signals as well as synchronous discharge in epileptic seizures.

In the acquired epilepsies, the seizure-prone state is considered to originate from a progressive series of molecular, cellular and circuit alterations evolving over time. In other words, epileptogenesis is regarded as a process that seems to be the antithesis of homeostasis, which is a fundamental property of animal physiology. In the past decades, however, “paradoxical” or “compensatory” responses to seizure activity have been demonstrated. Intriguingly, we found an additional “paradoxical” response to seizure activity in our study. Although abDGCs born at the acute phase after animals being fully kindled increased the duration of later seizures, they were functionally inhibited during GS expression with unclear mechanisms. Apart from numerous studies indicated a pro-epileptic role of abDGCs, Jakubs et al., also found that newborn abDGCs in the epileptic brain exhibited reduced excitability using whole-cell patch-clamp recordings.^30^ In epileptic rats, loss of GABAergic interneuron initially reduces numbers of GABAergic synapses with DGCs; however, synaptogenesis by surviving interneurons overshoots control levels subsequently.^58^ In our study, using a dual-virus tracing strategy combined with IHC, we also found that the percentage of GABAergic inputs (mainly from the local hippocampus subregions) in abDGCs generated acutely after seizures significantly increased. Additionally, as was reported, after SE, although parvalbumin and somatostatin interneuron inputs onto ebDGCs were significantly diminished, somatostatin interneuron inputs onto abDGCs were maintained.^33^ It is possible that the abDGCs rather than ebDGCs are inhibited by receiving inputs from the GABAergic neuron, including somatostatin interneurons, to help prohibit the seizure onset and progression, serving as one of the “compensatory” homeostatic mechanisms actively engaging in the epileptic brain. How the upstream feedback or feedforward circuit modulate abDGC is an important unsolved question. This unexpected, but important finding may help to replenish our understanding of hippocampal neurogenesis and epilepsy, that is, homeostatic mechanisms are unable to re-establish neuronal activity and prohibit the seizure onset and progression.

Further, different from previous findings by Zhou et al., our results reveal that repeated modulation of the above-mentioned homeostatic mechanisms may lead to change of synaptic plasticity which underlies the mechanism of the long-lasting anti-seizure effect of abDGCs compared with developmentally generated counterparts. Notably, in hippocampal kindling model, we found that increased percentage of animals were found to maintain their increased or decreased ADD and GSD post photo-stimulation after receiving 7 photo-stimulations, compared with 3 photo-stimulations. Meanwhile, in KA-induced epilepsy model, the seizure duration and frequency of most animals didn’t return to baseline level after the withdrawal of CNO, while the protective effect increasing with time during CNO treatment. This is consistent with the previous finding that a single ablation of adult neurogenesis was sufficient to generate suppressive effects which last for nearly 1 year as was reported.^23^ Since epilepsy is generally a life-long disease with debilitating recurrent seizures, it is necessary for us to develop long-lasting modalities to alleviate chronic seizures. Compared with ebDGCs, abDGCs may be an ideal choice to satisfy the need of long-lasting anti-seizure effects. Indeed, abDGCs even at an older developmental stage (2–3 months of age) still have the potential for experience-dependent plasticity, whereas developmentally generated neurons cannot be shaped under similar conditions.^59^ Taken together, the long-lasting effect significantly reinforce the therapeutic relevance of abDGCs as a future target for epilepsy.

## Materials and methods

### Animals

All animals (C57BL/6 mice, male, 20–25 g) used in this study were healthy and not involved in any previous test or drug treatment. Before surgery, they were maintained in groups in cages with a 12-h light/dark cycle (light on from 8:00 to 20:00) where they had free access to water and food. After surgery, they were individually housed to facilitate their recovery and to reduce the failure rate of cannula and electrode implantation. All behavior experiments were conducted during the daytime between 9:00 and 18:00. Meanwhile, all experiments were performed complying with the ethical guidelines of the Zhejiang University Animal Experimentation Committee and were in accordance with the National Institutes of Health Guide for the Care and Use of Laboratory Animals.

### Virus

The pUX-cre constructs were produced using a murine leukemia retroviral vector to express Cre recombinase as previous studies;^25,36^ by combining it with the use of Cre-inducible recombinant adeno-associated virus (AAV), selective expression can be conferred to proliferating cells. For Ca^2+^ fluorometric monitoring of abDGCs, a viral cocktail (1:1, 0.6 μL) of AAV-EF1a-DIO-GCaMP6s and pUX-Cre (retrovirus) was injected stereotactically into the DG of C57BL/6 mice (named *pUX-GCaMP6s* mice). Similarly, for selectively optogenetic activation or inactivation of abDGCs, a viral cocktail (1:1, 0.6 μL) of AAV-EF1a-DIO-ChR2-EYFP / AAV-hSyn-DIO-ChrimsonR-mCherry / AAV-CAG-FLEX-ArchT-GFP and pUX-Cre was stereotactically injected into the DG (named *pUX-ChR2* / *pUX-ChrimsonR / pUX-Arch* mice). Alternatively, another retrovirus pROV-EF1a-ChR2-mCherry (1.0 μL) was also used to “birthdate” and optogenetically activate abDGCs. To chemogenetically inactivate them, AAV-EF1a-DIO-hM4Di-mCherry was injected simultaneously with pUX-Cre. To selectively modulate ebDGCs, which had matured by the time of seizure induction, mice were stereotactically injected with 0.3-μL AAV-CaMKIIα-ChR2-mCherry, AAV-CaMKIIα-ArchT-EGFP or AAV-CaMKIIα-hM4Di-mCherry. Besides, AAV-CaMKIIα-GCaMP6s-EGFP was used for Ca^2+^ fluorometric monitoring of ebDGCs. AAV-CaMKIIα-GFP and AAV-Ef1a-DIO-mCherry were used as control viruses. The pUX-cre was kindly provided by Prof. Yan Gu from Zhejiang University. AAV-hSyn-DIO-ChrimsonR-mCherry was purchased from Brain VTA Corp., ltd. (Wuhan, China). In retrograde tracing experiments, retrograde tracing rabies virus was used to reveal the direct presynaptic inputs of ebDGCs and abDGCs. AAV-CaMKIIα-cre (0.2 μL) and pUX-cre (0.5 μL) were seperately combined with AAV2/9-hSyn-FLEX-mCherry-2A-TVA-2A-RvG-WPRE-pA (0.2 μL) viruses to allow the initial infection of DG starter neurons and RG coding for the rabies virus envelope glycoprotein as well as the follwing trans-synaptic spread of the virus. Four weeks later, the same location was microinjected with RV-ENVA-ΔG-GFP (0.1 μL). All viruses were commercially available from OBiO Technolog Corp., Ltd. (Shanghai, China), except for the retrograde tracing rabies viruses, which was pruchased from Taitool Bioscience Co., Ltd (Shanghai, China).

### Electrode/fiber implantation and viral injection surgery

Similar to our previous studies,^34,35^ under sodium pentobarbital anesthesia (50 mg/kg, i.p.), mice were mounted in a stereotaxic apparatus and bipolar stainless-steel Teflon-coated electrodes (0.125 mm in diameter; A.M. Systems, USA) were implanted into right ventral hippocampus for kindling stimulation and EEG recording; Meanwhile, a customized cannula (Catalog No.62003, RWD Life Science, China) was implanted into right DG for virus injection, optical stimulation and intra-DG drug injection. Viruses were injected with a 1-μL microliter syringes controlled by an injection pump (Micro 4, WPI Inc., USA) through the implanted cannula at the rate of 100 nl/min. The precise loactions of each brain region used in our experiements are listed as follwing, right ventral hippocampus (AP, −2.9 mm; ML, −3.2 mm; DV, −3.2 mm), right hippocampus (AP, −2.0 mm; ML, −1.3 mm; DV, −1.6 mm) and DG (AP, −2.0 mm; ML, −1.1 mm; DV, −1.9 mm), which are determined according to the atlas of Franklin and Paxinos for mice.^60^ All locations of electrode and cannula implantation as well as viral expression were histologically verified after experiments in all animals.

### Hippocampal kindling model

After about 7–10 days of recovery, mice were performed with rapid hippocampal kindling acquisition as our previous studies.^34,35^ All mice received 10 kindling stimulations delivered by a constant-current stimulator daily every 30 min. The parameter for kindling stimualtion is monophasic square-wave pulses, 20 Hz, 1 ms/pulse, 40 pulses, 400μA. EEGs were simultaneously recorded with a Neuroscan system (Compumedics, Australia). Seizure stage (according to Racine scale,^61^) latency to GS, ADD and GSD (according to EEG analysis) were recorded and evaluated by trained investigators who were unaware of the design of experiments. Once a mouse is fully kindled (experienced consecutive 3-times stage 5 seizures), it remains displaying stable GS in response to kindling stimulation for months.^62^ Specifically, data from the initial 3 stimulations were used to determine the basal level of seizure severity (pre). Different interventions, including optogenetic, chemogenetic or pharmacological modulations were used during the next 3 or 7 stimulations, and finally followed by an additional 3 stimulations to evaluate whether there exists persisting effect (post).

### Light stimulation and Pharmacology

Light was delivered through a 200 μm-diameter optic fiber connected to the laser (BL473T3-050 or YL589T3-050, Shanghai Laser & Optics Century Co., Ltd., China) and triggered by a Master-8 stimulator. The stimulation parameter of blue light (473 nm) we used is 20 Hz, 10 ms/pulse and 600 pulses, 5 mW. On the other hand, continuous 30 s yellow light (589 nm, direct current, 5 mw) stimulation was applied. Light stimulation was given immediately after the cessation of kindling stimulation to mimic close-loop stimulation pattern.

Pharmacological drugs were focally injected into the DG of freely moving mice through the cannula with an inserted injection needle (Catalog No.62004, RWD Life Science, China) connected to a 1-µL microliter syringes by PE tubing (Catalog No.62320, RWD life science, China). The injection duration for drugs was 2 min and the needle was left in place for another 5 min before withdrawal. For glutamatergic receptor antagonists, we used a blocker cocktail (1:1, 0.5 μL) of 10 μM CNQX and 25 μM D-AP5 dissolved in saline. The drugs were injected 5 min before the insertion of optic fiber to deliver blue light. Control animals received only glutamatergic receptor antagonists to test whether glutamatergic receptor alone had effect on seizures.

For chemogenetic studies, all kindled mice were i.p. injected with CNO (3.0 mg/kg, Abcam, ab141704) 0.5 h before the first stimulation during the intervention period. Control animals were treated identically with CNO; however, they were injected with AAV-CamKIIα-GFP instead of AAV-CamKIIa-hM4Di-mCherry.

### Fiber photometry

The calcium signals of GCaMP fluorescence was carried out in *pUX-GCaMP6* mice using the fiber photometry system (Nanjing Thinkertech, China) as our previous studies.^34,35^ During the GS expression, EEG was recorded simultaneously with the photometry data of calcium signals. To facilitate further analysis, photometry data was then exported to MATLAB Mat files. Then, we segmented the data according to individual trails of kindling stimulations and ultimately derived the values of fluorescence change (ΔF/F) by calculating (F − F_0_)/F_0_, which were presented with average plots and individual maximum plots in our figures.

### Hippocampal EEG recording and analysis

Hippocampal EEGs were recorded and analyzed using Scan 4.5 system (Compumedics Ltd, Australia) according to previous studies.^34,63^ Raw EEG signals were sampled at 1 kHz. EEG epochs with obvious artifacts were rejected by visual inspection. Other remaining EEG epochs were digitally band-pass filtered (0.3–100 Hz), divided into consecutive 4 s time series and then used for spectral analysis utilizing the fast Fourier transform (FFT). Then, the frequency bins were averaged within six frequency bands between 0 and 100 Hz: delta (0.5–4 Hz), theta (4–8 Hz), alpha (8–12 Hz), beta (12–30 Hz), as well as gamma (30–100 Hz).

### Single-unit recording and analysis

Neuronal activities were recorded in urethane anesthetized kindled *pUX-ChR2* mice and analyzed using Cerebus acquisition system (Blackrock Microsystems, AUS) according to previous studies.^34,35,46,47^ Recording was made with a bundle microelectrode of eight 25-µm nicherome wires coated by formvar (Cat No.761500, AM-system, USA), which was stuck with an optical fiber, with the tip of the electrodes extending 500 μm above the fiber. Meanwhile, the bundle microelectrode was grounded to a screw fixed above the cerebellum and subsequently referenced online against a wire within the same brain region. Neuronal activity was sampled at 30 kHz, high-pass filtered at 250 Hz and sorted online. When the optic fiber was descended into the DG and stable spikes of putative DGCs were detected, pulsed laser (20 Hz, 10 ms width, 1 s on/4 s off cycles) was delivered to test neuronal responsiveness to optogenetic activation of abDGC. Based on previous studies,^44,45,64^ the feature of putative DGCs were identified as following: low firing rate (≤2 Hz) and large spike widths (with ≥0.25 mV). Based on kinetic responses to blue light,^46,47^ our recorded responsive DGCs could be categorized into two groups: ChR2-expressing abDGCs (with a short latency, <4 ms) and downstream ebDGCs.

### Intra-hippocampal KA chronic epilepsy model

KA (0.25 μg in 0.5 μl saline) was stereotaxically injected into the right hippocampus with the same coordinates as our previous studies.^34,35^ One week later, the surviving mice were injected a viral cocktail (AAV-EF1a-DIO-hM4Di-mCherry and pUX-cre) into the dorsal DG to label abDGCs. About 2 months after injection, the surviving mice were implanted with electrodes into the right ventral hippocampus for EEG recording. After recovery from surgery, freely moving mice underwent EEG monitoring using a PowerLab system (AD Instruments, Australia) at a sampling rate of 1 kHz for 5 days to establish baseline seizure frequency (baseline). Mice received daily CNO (i.p., 1 mg/kg) for five consecutive days to test the effect of chemogenetic inactivation of abDGCs (CNO), compared with 5-day baseline level (baseline) and additional 5-day recordings at post-CNO level (post). The definition of spontaneous seizure is: regular spike clusters with a duration of ≥10 s, spike frequency ≥2 Hz and amplitude ≥ 3-times baseline EEG. The number and duration of SRS were seperately calculated over an 8-h period in total each day during the experiment. Control animals were injected with AAV-Ef1a-DIO-EYFP and pUX-cre after KA injection and treated identically in other aspects.

### Immunohistochemistry

IHC was performed similar to our previous studies,^34,35^ except for BrdU. Specially, for immunohistochemical detection of incorporated BrdU, all mice received a single series of 4 injections of 50 mg/kg BrdU (Sigma B5002, dissolved in saline) with 2 h interval to label mitotically active cells, and were perfused 24 h after the last injection; tissue was incubated in 2 N HCL at 37°C for 0.5 h, and then sections were washed in 0.1 M boric acid (pH 8.5) for 5 min*3 times to achieve DNA denaturation. We processed sections for immunofluorescence for PROX1 (1:400, Abcam, ab101851), c-fos (1:200, SYSY, 226005), DCX (1:400, Abcam, ab18723), GABA(1:600, Sigma, A2052) as well as DNA-denaturated BrdU (1:400, Abcam ab1893) by incubating the sections with primary antibodies diluted in phosphate-buffered saline with 0.15% Triton X-100 overnight at 4 °C. Then, the sections were incubated with secondary antibodies Alexafluor 488 (ab150181, Abcam), 594 (712-005153, Jackson Immunoresearch) or 647 (ab150075, Abcam/A21450, Invitrogen) at room temperature for 2 h. Finally, immunofluorescence was assessed using confocal microscope (Olympus FV1200).

Image analysis and quantification were conducted using Image J software (version 1.52a). For BrdU calculation experiments, one section from every twelfth 20-μm coronal section throughout the DG was chosen and manually scored by a blinded examiner for BrdU-immunostained nuclei. For colocalization experiments, for example, to validate the specificity of dual-virus strategies in labeling abDGCs with ChR2, we calculated the mean number of cells that were both ChR2^+^ and DCX^+^ in three representative coronal brain slices (anterior, intermediate and posterior). Selected immunofluorescence images were analyzed by orthogonal reconstructions from z-series (z-step, 1.5 μm) to derive the number of double-labeling cells. Other colocalization evaluation was conducted using the same method.

### In vitro electrophysiology

To obtain acute DG slices, *pUX-ChR2* mice which were injected with viruses 3 days after being fully kindled were decapitated and the brains were dissected immediatley and immersed in ice-cold oxygenated (95% O_2_ and 5% CO_2_) artificial cerebrospinal fluid (ACSF) containing in mM: 1.3 NaH_2_PO_4_, 110 choline chloride, 25 NaHCO_3_, 2.5 KCl, 7 MgCl_2_, 1.3 ascorbic acid, 0.5 CaCl_2_, 20 D-glucose, and 0.6 Na-pyruvate, pH 7.3. We cut coronal slices containing DG at 300 µm thickness using a vibratome (VT1000S, Leica Instruments Ltd) and incubated them at 37 °C. Then, an experimental brain slice was transferred to a submersion-recording chamber that is continuously perfused with recording oxygenated solution (containing in mM: 125 NaCl, 1.3 NaH_2_PO_4_, 2.5 KCl, 25 NaHCO_3_, 1.3 MgCl_2_, 1.3 ascorbic acid, 2 CaCl_2_, 10 D-glucose, and 0.6 Na-pyruvate). Whole-cell patch clamp recordings were performed using an EPC10 patch-clamp amplifier (HEKA Instruments) at a sampling rate of 1 kHz, low-pass filtered at 3 kHz. To record light evoked EPSCs, recording pipettes (tip resistances, ~ 4–8 MΩ) were filled with a solution containing in mM: 5 NaCl, 140 CsCl, 10 HEPES, 0.2 EGTA, 0.3 Na_3_GTP, 10 Na_2_-phosphocreatine, 2 Mg-ATP, 5 QX314, pH 7.2. The recorded neurons were holding at −70 mV. The EPSCs were isolated by addition of bicuculline (20 μM) to block inhibitory transmission mediated by GABA. Light pulse at the selected parameter (1 Hz, 10 ms, 5 pulses, 2 mW) was controlled with EPC10 patch-clamp amplifier.

### Statistics

All data are presented as the mean ± S.E.M. Statistical method and number of experimental replicates (*n*) is indicated in each figure legend. Statistical comparisons were performed using Prism (version 7.0). A two-tailed *P* < 0.05 was considered statistically significant.

## Data and materials availability

All data needed to evaluate the conclusions in the paper are present in the paper and/or the Supplementary Materials. Additional data related to this paper are available from the corresponding author upon request.

## Supplementary information

Supporting Materials

## References

[1] Thijs RD, Surges R, O’Brien TJ, Sander JW (2019). Epilepsy in adults. Lancet.

[2] Collaborators, G.B.D.E. (2019). Global, regional, and national burden of epilepsy, 1990–2016: a systematic analysis for the Global Burden of Disease Study 2016. Lancet Neurol..

[3] Engel J (2001). Mesial temporal lobe epilepsy: what have we learned?. Neuroscientist.

[4] Wang Y, Chen Z (2019). An update for epilepsy research and antiepileptic drug development: Toward precise circuit therapy. Pharmacol. Ther..

[5] Jobst BC, Cascino GD (2015). Resective epilepsy surgery for drug-resistant focal epilepsy: a review. JAMA.

[6] Perucca P, Gilliam FG (2012). Adverse effects of antiepileptic drugs. Lancet Neurol..

[7] Anacker C, Hen R (2017). Adult hippocampal neurogenesis and cognitive flexibility - linking memory and mood. Nat. Rev. Neurosci..

[8] Aimone JB (2014). Regulation and function of adult neurogenesis: from genes to cognition. Physiol. Rev..

[9] Huckleberry KA (2018). Dorsal and ventral hippocampal adult-born neurons contribute to context fear memory. Neuropsychopharmacology.

[10] Danzer SC (2012). Depression, stress, epilepsy and adult neurogenesis. Exp. Neurol..

[11] Chen L, Wang Y, Chen Z (2019). Adult neurogenesis in epileptogenesis: An update for preclinical finding and potential clinical translation. Curr. Neuropharmacol..

[12] Parent JM (1997). Dentate granule cell neurogenesis is increased by seizures and contributes to aberrant network reorganization in the adult rat hippocampus. J. Neurosci..

[13] Scott BW, Wang S, Burnham WM, De Boni U, Wojtowicz JM (1998). Kindling-induced neurogenesis in the dentate gyrus of the rat. Neurosci. Lett..

[14] Jessberger S (2007). Seizure-associated, aberrant neurogenesis in adult rats characterized with retrovirus-mediated cell labeling. J. Neurosci..

[15] Scharfman HE, Goodman JH, Sollas AL (2000). Granule-like neurons at the hilar/CA3 border after status epilepticus and their synchrony with area CA3 pyramidal cells: functional implications of seizure-induced neurogenesis. J. Neurosci..

[16] Overstreet-Wadiche LS, Bromberg DA, Bensen AL, Westbrook GL (2006). Seizures accelerate functional integration of adult-generated granule cells. J. Neurosci..

[17] Ribak CE, Tran PH, Spigelman I, Okazaki MM, Nadler JV (2000). Status epilepticus-induced hilar basal dendrites on rodent granule cells contribute to recurrent excitatory circuitry. J. Comp. Neurol..

[18] Shapiro LA, Korn MJ, Ribak CE (2005). Newly generated dentate granule cells from epileptic rats exhibit elongated hilar basal dendrites that align along GFAP-immunolabeled processes. Neuroscience.

[19] Jung KH (2004). Continuous cytosine-b-D-arabinofuranoside infusion reduces ectopic granule cells in adult rat hippocampus with attenuation of spontaneous recurrent seizures following pilocarpine-induced status epilepticus. Eur. J. Neurosci..

[20] Raedt R (2007). Radiation of the rat brain suppresses seizure-induced neurogenesis and transiently enhances excitability during kindling acquisition. Epilepsia.

[21] Iyengar SS (2015). Suppression of adult neurogenesis increases the acute effects of kainic acid. Exp. Neurol..

[22] Pun RY (2012). Excessive activation of mTOR in postnatally generated granule cells is sufficient to cause epilepsy. Neuron.

[23] Cho KO (2015). Aberrant hippocampal neurogenesis contributes to epilepsy and associated cognitive decline. Nat. Commun..

[24] Zhou QG (2019). Chemogenetic silencing of hippocampal neurons suppresses epileptic neural circuits. J. Clin. Invest..

[25] Gu Y (2012). Optical controlling reveals time-dependent roles for adult-born dentate granule cells. Nat. Neurosci..

[26] Nakashiba T (2012). Young dentate granule cells mediate pattern separation, whereas old granule cells facilitate pattern completion. Cell.

[27] Lybrand ZR (2021). A critical period of neuronal activity results in aberrant neurogenesis rewiring hippocampal circuitry in a mouse model of epilepsy. Nat. Commun..

[28] Spigelman I (1998). Dentate granule cells form novel basal dendrites in a rat model of temporal lobe epilepsy. Neuroscience.

[29] McCloskey DP, Hintz TM, Pierce JP, Scharfman HE (2006). Stereological methods reveal the robust size and stability of ectopic hilar granule cells after pilocarpine-induced status epilepticus in the adult rat. Eur. J. Neurosci..

[30] Jakubs K (2006). Environment matters: synaptic properties of neurons born in the epileptic adult brain develop to reduce excitability. Neuron.

[31] Murphy BL (2011). Heterogeneous integration of adult-generated granule cells into the epileptic brain. J. Neurosci..

[32] Walter C, Murphy BL, Pun RY, Spieles-Engemann AL, Danzer SC (2007). Pilocarpine-induced seizures cause selective time-dependent changes to adult-generated hippocampal dentate granule cells. J. Neurosci..

[33] Du X, Zhang H, Parent JM (2017). Rabies tracing of birthdated dentate granule cells in rat temporal lobe epilepsy. Ann. Neurol..

[34] Wang Y (2019). Direct septum-hippocampal cholinergic circuit attenuates seizure through driving somatostatin inhibition. Biol. Psychiatry.

[35] Chen B (2020). A disinhibitory nigra-parafascicular pathway amplifies seizure in temporal lobe epilepsy. Nat. Commun..

[36] van Praag H (2002). Functional neurogenesis in the adult hippocampus. Nature.

[37] Ge S (2006). GABA regulates synaptic integration of newly generated neurons in the adult brain. Nature.

[38] Toni N (2008). Neurons born in the adult dentate gyrus form functional synapses with target cells. Nat. Neurosci..

[39] Johnston S (2021). AAV ablates neurogenesis in the adult murine hippocampus. Elife.

[40] McHugh SB (2022). Adult-born dentate granule cells promote hippocampal population sparsity. Nat. Neurosci..

[41] Wang Y (2017). Depolarized GABAergic signaling in subicular microcircuits mediates generalized seizure in temporal lobe epilepsy. Neuron.

[42] Hattiangady B, Rao MS, Shetty AK (2004). Chronic temporal lobe epilepsy is dentate neurogenesis in the adult associated with severely declined hippocampus. Neurobiol. Dis..

[43] Kron MM, Zhang H, Parent JM (2010). The developmental stage of dentate granule cells dictates their contribution to seizure-induced plasticity. J. Neurosci..

[44] Jung MW, McNaughton BL (1993). Spatial selectivity of unit activity in the hippocampal granular layer. Hippocampus.

[45] Leutgeb JK, Leutgeb S, Moser MB, Moser EI (2007). Pattern separation in the dentate gyrus and CA3 of the hippocampus. Science.

[46] Wei P (2015). Processing of visually evoked innate fear by a non-canonical thalamic pathway. Nat. Commun..

[47] Yang Y (2016). Opposite monosynaptic scaling of BLP-vCA1 inputs governs hopefulness- and helplessness-modulated spatial learning and memory. Nat. Commun..

[48] Kahn JB, Port RG, Yue C, Takano H, Coulter DA (2019). Circuit-based interventions in the dentate gyrus rescue epilepsy-associated cognitive dysfunction. Brain.

[49] Kirschen GW (2017). Active dentate granule cells encode experience to promote the addition of adult-born hippocampal neurons. J. Neurosci..

[50] Gray WP, Sundstrom LE (1998). Kainic acid increases the proliferation of granule cell progenitors in the dentate gyrus of the adult rat. Brain Res..

[51] Lacefield CO, Itskov V, Reardon T, Hen R, Gordon JA (2012). Effects of adult-generated granule cells on coordinated network activity in the dentate gyrus. Hippocampus.

[52] Ikrar T (2013). Adult neurogenesis modifies excitability of the dentate gyrus. Front. Neural Circuits.

[53] Drew LJ (2016). Activation of local inhibitory circuits in the dentate gyrus by adult-born neurons. Hippocampus.

[54] Temprana SG (2015). Delayed coupling to feedback inhibition during a critical period for the integration of adult-born granule cells. Neuron.

[55] Luna VM (2019). Adult-born hippocampal neurons bidirectionally modulate entorhinal inputs into the dentate gyrus. Science.

[56] Heinemann U (1992). The dentate gyrus as a regulated gate for the propagation of epileptiform activity. Epilepsy Res. Suppl..

[57] Lothman EW, Stringer JL, Bertram EH (1992). The dentate gyrus as a control point for seizures in the hippocampus and beyond. Epilepsy Res. Suppl..

[58] Thind KK (2010). Initial loss but later excess of GABAergic synapses with dentate granule cells in a rat model of temporal lobe epilepsy. J. Comp. Neurol..

[59] Lemaire V (2012). Long-lasting plasticity of hippocampal adult-born neurons. J. Neurosci..

[60] Paxinos, G., Franklin, K.B.J. & Franklin, K.B.J. *The Mouse Brain in Stereotaxic Coordinates* (Academic Press, San Diego, 2001).

[61] Racine RJ (1972). Modification of seizure activity by electrical stimulation. II. Motor seizure. Electroencephalogr. Clin. Neurophysiol..

[62] Goddard GV, McIntyre DC, Leech CK (1969). A permanent change in brain function resulting from daily electrical stimulation. Exp. Neurol..

[63] Xu Z (2013). Polarity-dependent effect of low-frequency stimulation on amygdaloid kindling in rats. Brain Stimul..

[64] Wiebe SP, Staubli UV (1999). Dynamic filtering of recognition memory codes in the hippocampus. J. Neurosci..

