# Supplementary material for: Adult-born neurons in critical period maintain hippocampal seizures via local aberrant excitatory circuits
Source: Signal Transduct Target Ther. 2023 Jun 7;8:225. doi: 10.1038/s41392-023-01433-4 (PMC10244397; doi:10.1038/s41392-023-01433-4)
Supplement: Supplementary file 1 — Supporting Materials [file 41392_2023_1433_MOESM1_ESM.docx]

Supplementary Materials for

**Adult-born Neurons in Critical Period Maintain Hippocampal Seizures via Local Aberrant Excitatory Circuits**

Liying Chen^1^†, Yingwei Xu^1^†, Heming Cheng^2^†, Zhongxia Li^2,3^, Nanxi Lai^1^, Menghan Li^2^, Yeping Ruan^2^, Yang Zheng^2^, Fan Fei^1,2^, Cenglin Xu^2^, Jiao Ma^1^, Shuang Wang^1^, Yan Gu^1^, Feng Han^4^, Zhong Chen^1,2^*, and Yi Wang^1,2,3^*

Correspondence to: chenzhong@zju.edu.cn (Z. Chen), wang-yi@zju.edu.cn (Y. Wang)

**This file includes:**

Figures. S1 to S11

**Other Supplementary Materials for this manuscript include the following:**

Data S1


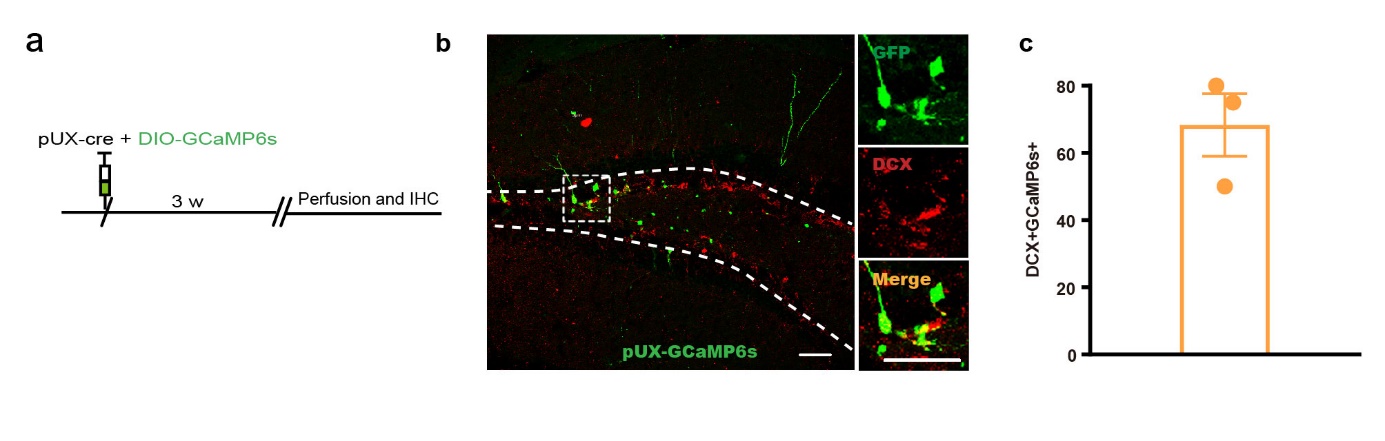


**Figure. S1.**

**Histochemical verification of GCaMP-expressing abDGCs labelled with retrovirus.** (**a-c**) Histochemical verification of GCaMP-expressing abDGCs labelled with retrovirus. (**a**) Experiment scheme (mice were perfused 3W post retrovirus injection). (**b**) Representative images of double immunostaining of GCaMP (GFP) with DCX (red) (bar = 50 μm). (**c**) Percentage of retrovirus labeled, immunochemically identified (DCX^+^) abDGCs (DCX^+^ GCaMP ^+^% = (DCX^+^ GCaMP ^+^) / GCaMP ^+^×100%; n =3, GCaMP ^+^ cells were from 3 non-kindled mice, 68.33% were DCX^+^).


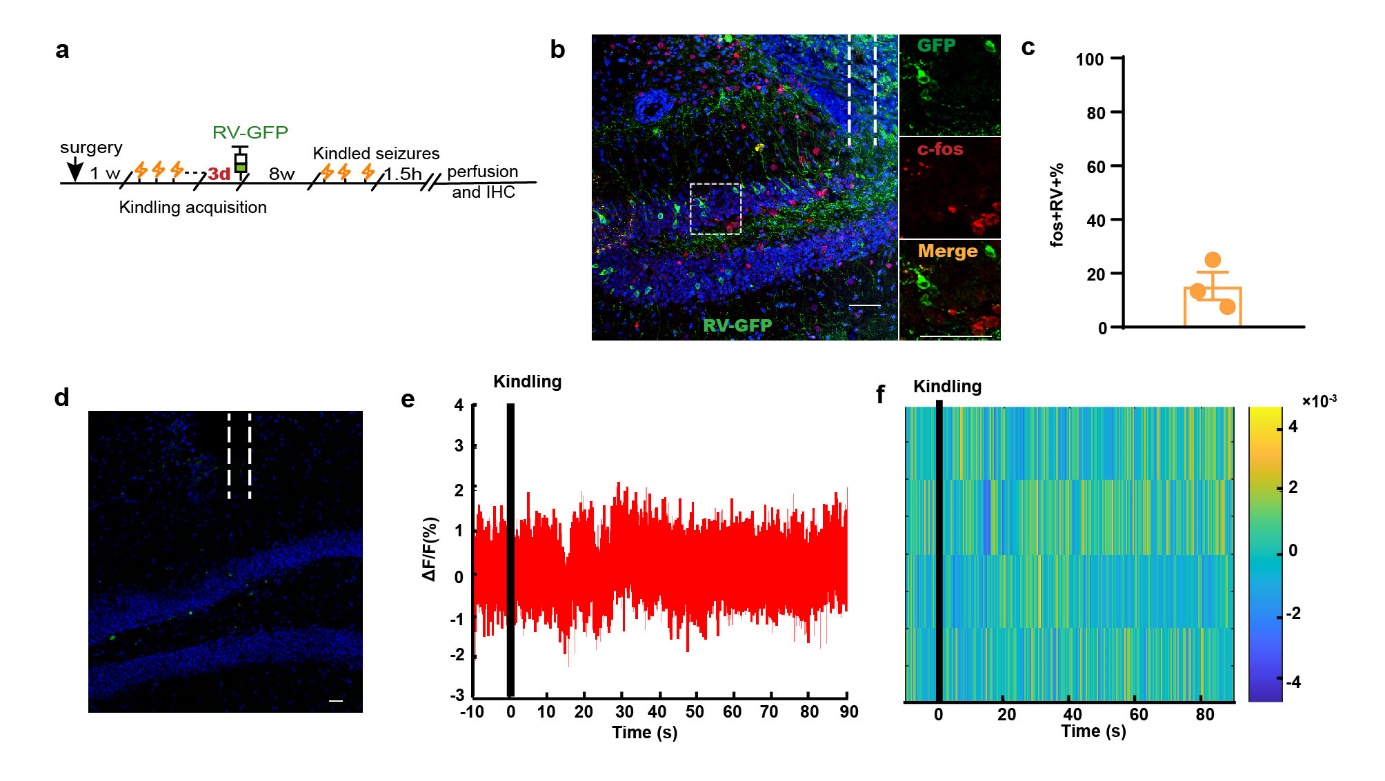


**Figure. S2.**

**abDGCs are not c-fos+ after hippocampal seizures.** (**a**) Schematic diagram of c-fos immunostaining. (**b**) Representative images of double immunostaining of retrovirus (GFP) with c-fos (red), right panels are enlarged images (bar = 50 μm). (**c**) Percentage of retrovirus labeled, immunochemically identified (c-fos+) activated abDGCs (fos+RV+%=（c-fos+RV+）/RV+×100%; n=3, RV+ cells were from 3 kindled mice, only 15.3% were c-fos+). Mice were perfused 1.5 after 3 times of fully-kindled seizrues. (**d-f**) No significant GCaMP fluorescence change is observed in opsin-negative animals. (**d**) Representative image of histochemical verification of GCaMP6s negative expression and placement of cannula (bar = 50 μm). (**e**) Mean fluorescence values during hippocampal seizures (n=4). (**f**) Fluorescence responses of each animal.

**
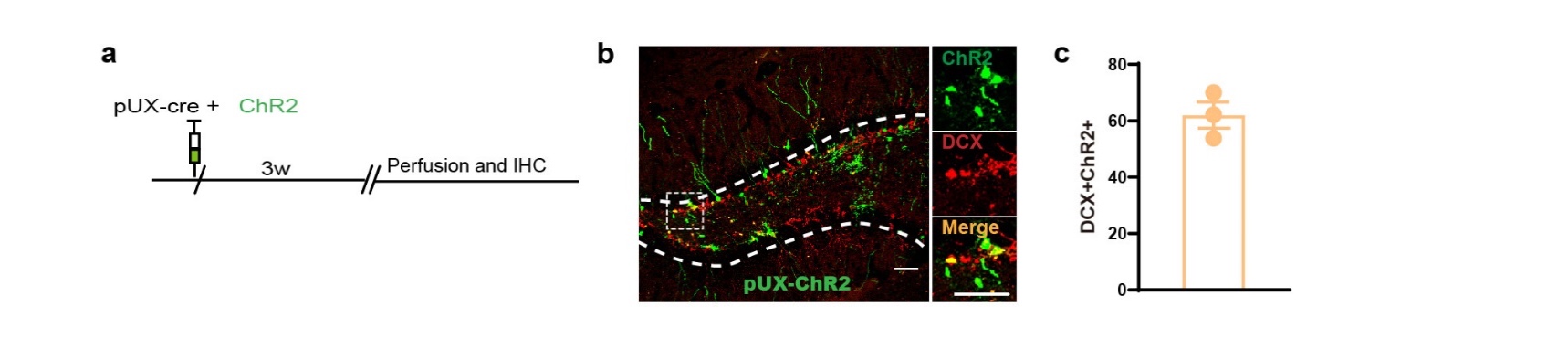
**

**Figure. S3.**

**Histochemical verification of ChR2-expressing abDGCs labelled with retrovirus.** (**a**) Experiment scheme (mice were perfused 3W post retrovirus injection). (**b**) Representative images of double immunostaining of ChR2 (GFP) with DCX (red) (bar = 50 μm). (**c**) Percentage of retrovirus labeled, immunochemically identified (DCX^+^) abDGCs (DCX^+^ ChR2 ^+^% = (DCX^+^ ChR2 ^+^) / ChR2 ^+^×100%; n=3, ChR2 ^+^ cells were from 3 non-kindled mice, 61.97% were DCX^+^).

**
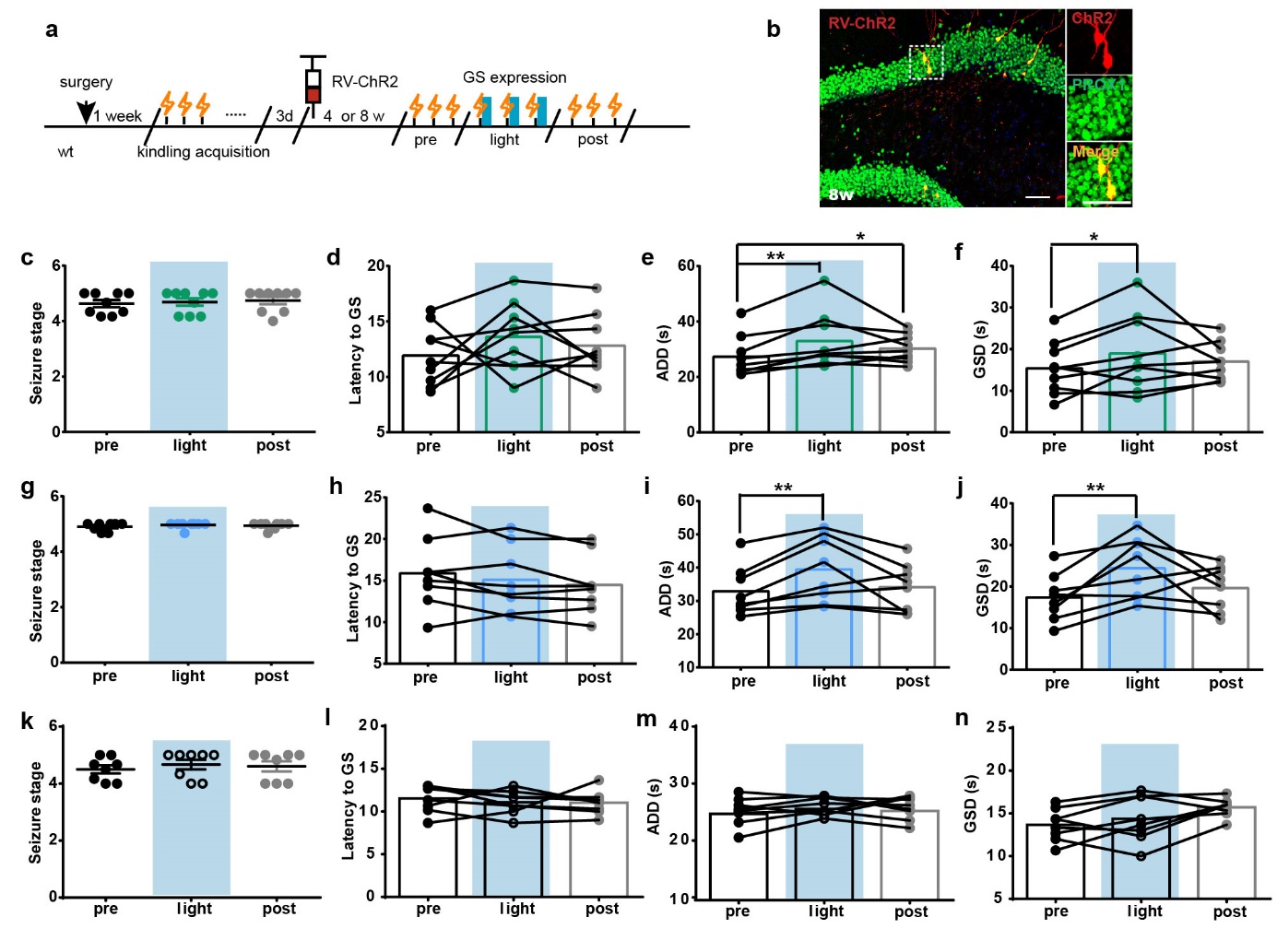
**

**Figure. S4.**

**Optogenetic activation of abDGCs by using RV-ChR2 extends seizure duration, while light delivery to opsin-negative controls has no effect on hippocampal seizures**. (**a**) Experiment scheme for optogenetic activation protocol with retrovirus pROV-EF1a-ChR2-mCherry in recurrent hippocampal seizures. (**b**) Histochemical verification of ChR2-expressing abDGCs in the DG. Right panels are double immunostaining of PROX1 (green) and ChR2-mcherry (red) in brain slices with 8-weeks-old abDGCs (bar = 50 μm) and the enlarged images (bar = 50 μm). (**c-f**) Effects of optogenetic activation of the 4-weeks-old abDGCs by using RV-ChR2 on the seizure stage (**c**), latency to GS (**d**), ADD (**e**) and GSD (**f**) during hippocampal seizures (n=9, **p<0.01, *p<0.05, Paired t-tests). (**g-j**) Effects of optogenetic activation of the 8-weeks-old abDGCs by using RV-ChR2 on the seizure stage (**g**), latency to GS (**h**), ADD (**i**) and GSD (**j**) during hippocampal seizures (n=8, **p<0.01, Paired t-tests). **(k-n)** Effects of light delivery to opsin-negative controls on seizure stage (**k**), latency to GS (**l**), ADD (**m**) and GSD (**n**) during hippocampal seizures (n=8).


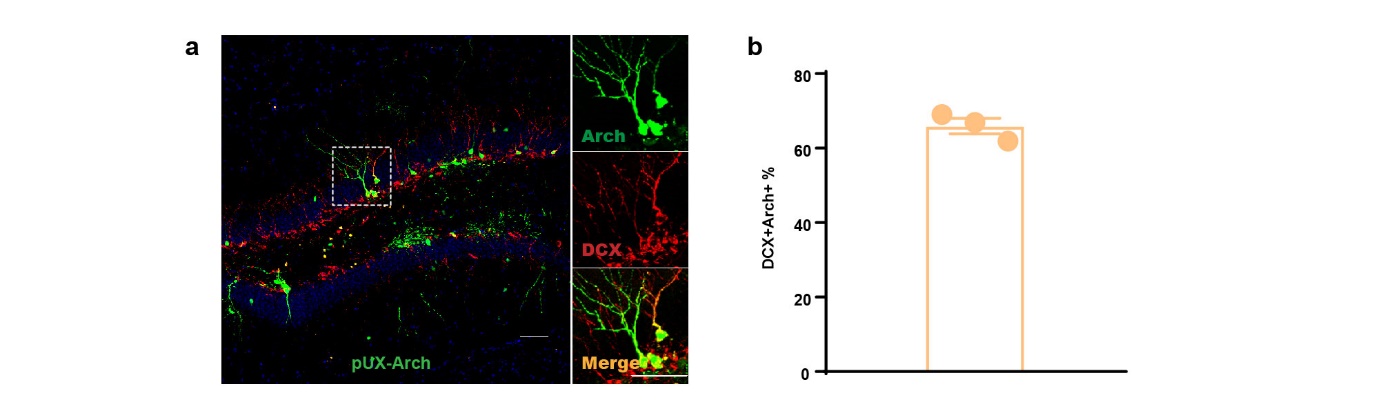


**Figure. S5.**

**Histochemical verification of Arch-expressing abDGCs labelled with retrovirus.** (**a**) Representative images of double immunostaining of Arch (GFP) with DCX (red). Mice were perfused 3W post retrovirus injection (bar = 50 μm). (**b**) Percentage of retrovirus labeled, immunochemically identified (DCX^+^) abDGCs (DCX^+^Arch^+^% = (DCX^+^Arch^+^) / Arch^+^×100%; n=3, Arch^+^ cells were from 3 non-kindled mice, 66.3% were DCX^+^).

**
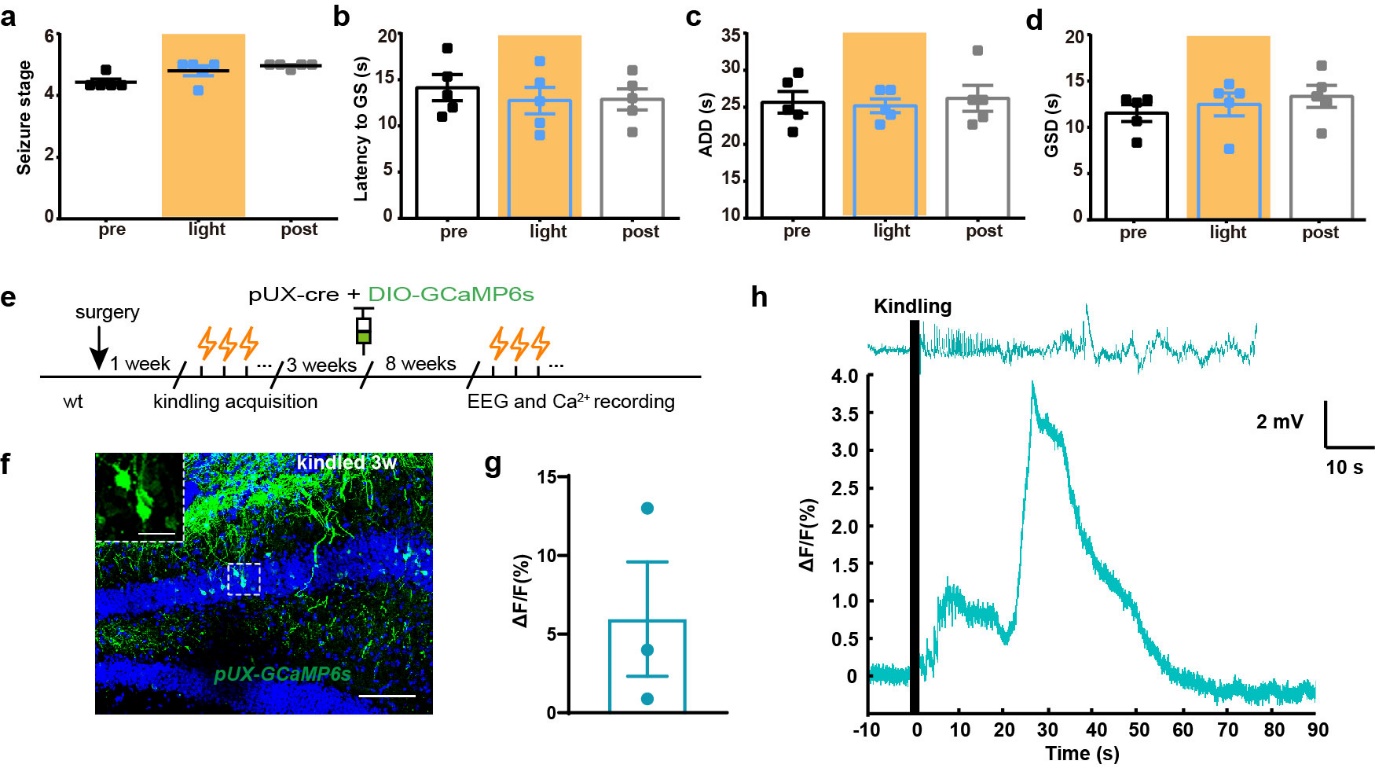
**

**Figure. S6.**

**Optogenetic inhibition of abDGCs born at 3 d before kindling exerts no significant effect on hippocampal seizures.** (**a-d**) Effects of optogenetic inhibition of 8-weeks-old abDGCs, labeled 3 days before kindling acquisition on seizure stage (**a**), latency to GS (**b**), ADD (**c**) and GSD (**d**) during hippocampal seizures (n=5). (**e**) Experimental paradigm of the Ca^2+^ fiber photometry experiment (virus was injected 3 weeks after mice were fully kindled). (**f**) Histochemical verification of GCaMP6s expression in abDGCs in coronal sections (bar = 50 μm) and the enlarged images (bar = 10 μm). (**g**) The statistical value of △F/F_0_ was shown for each mouse in the pUX*-GcaMP6s* group. (**h**) Representative GCaMP signal of abDGCs aligning with EEG recording during hippocampal seizures.


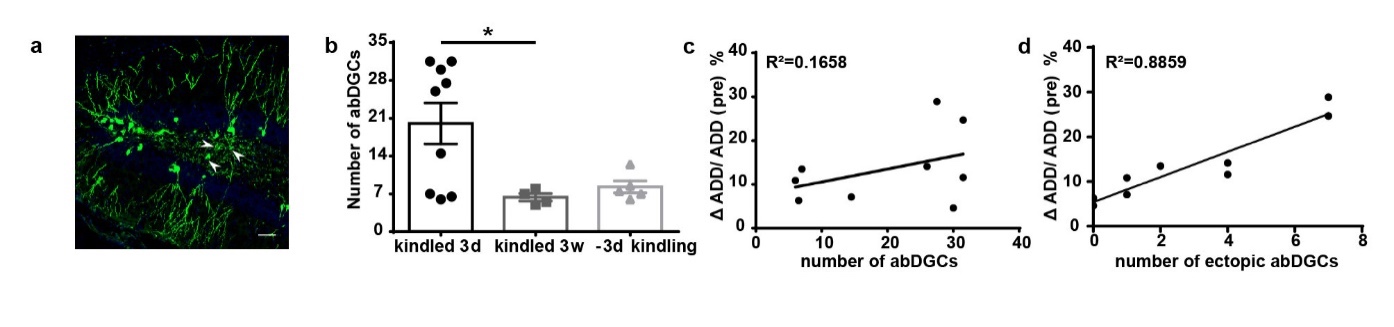


**Figure. S7.**

**The seizure-modulating effect was proportional to the number of ectopic abDGCs. (a)** Representative images of labelled abDGCs. White arrow indicates the ectopic abDGCs which are located ectopically in the hilus (bar=50 μm). **(b)** Calculation of the number of labelled abDGCs born at different timepoints. Labelled abDGCs born at 3d after fully kindled were significantly more compared with 3 weeks after (kindled 3d n=9, kindled 3w n=4, kindled -3d n=5; *p<0.05, One-way ANOVA followed by *post hoc* Dunnett test). **(c)** Correlational analysis between number of labeled cells (kindled 3d) and seizure-modulating effect. There was no significant correlation between number and effect (R^2^=0.1658, P=0.2766, Pearson correlation analysis was used) (n=9, 4 from pux-ChR2 mice, 5 from pux-Arch mice). Seizure-modulating effect was represented by **Δ**ADD/ADD (pre); for pux-ChR2 mice, **Δ**ADD was calculated as ADD(light)-ADD(pre), for pux-Arch mice, **Δ**ADD was calculated as ADD(pre) - ADD(light). **(d)** Correlational analysis between number of labeled ectopic abDGCs (kindled 3d) and seizure-modulating effect (R^2^=0.8859, P=0.0002, Pearson correlation analysis was used) (n=9, 4 from pux-ChR2 mice, 5 from pux-Arch mice).

**
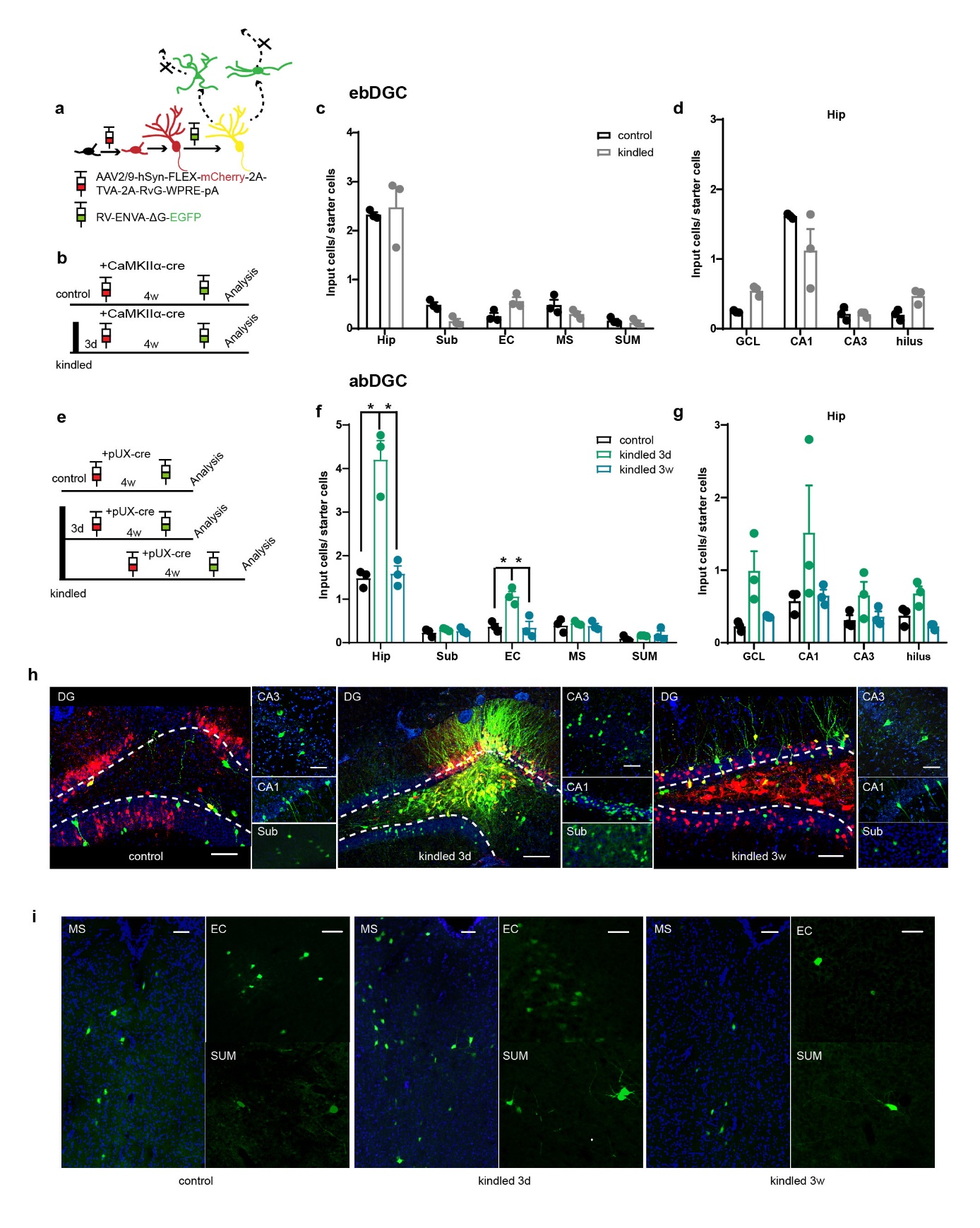
**

**Figure. S8.**

**Retrograde tracing of ebDGCs, abDGCs generated at acute and chronic phase after kindling-induced seizures.** (**a**) Experimental schematic showing the experimental design for rabies virus-mediated retrograde tracing. (**b**) Experimental schematic showing mapping of neuronal connectivity of ebDGCs. Control: non-kindled mice were injected with CamKⅡ-cre and AAV2/9-hSyn-FLEX-mCherry-2A-TVA-2A-RvG-WPRE-pA viruses; RV-ENVA-ΔG-GFP was injected at the same site after 4 weeks. Kindled: kindled mice were injected with CamKⅡ-cre and TVA-mCherry viruses; RV-GFP was injected after 4 weeks. Mice were perfused at 7 days after the injection of RV-GFP. (**c**) Connectivity ratios of input cells to starter cells of ebDGCs are shown, seperately for hippocampus (Hip), subiculum (Sub), entorhinal cortex (EC), medial septum (MS), as well as supramammillary nucleus (SUM). (**d**) Connectivity ratios of ebDGCs in different subregions of hippocampus are shown, seperately for granular cell layer (GCL), CA1, CA3 and hilus. (**e**) Experimental schematic showing mapping of neuronal connectivity of abDGCs generated at both acute and chronic phase after kindling. (**f**) Connectivity ratios of abDGCs are shown, seperately for Hip, Sub, EC, MS and SUM (n=3, *p<0.05, Two-way ANOVA followed by *post hoc* Dunnett test). (**g**) Connectivity ratios of abDGCs in different subregions of hippocampus are shown, seperately for GCL, CA1, CA3 and hilus. (**h**) Representative images of the TVA-mCherry and ENVA-GFP doubled-labeled starter cells in the DG injected with retrograde tracing virus (bar=50 μm). Rabies labeled GFP-expressing input cells are seen in the DG of non-kindled mice injected with CamKⅡ-cre and TVA-mCherry (close to the injection sites; left panel, bar= 50 μm) as well as in the CA1, CA3 and Sub (distant from the injection sites; right panel, bar= 50 μm). (**i**) Representative images of the ENVA-GFP labelled input cells seperately in the MS, EC and SUM (bar= 50 μm).


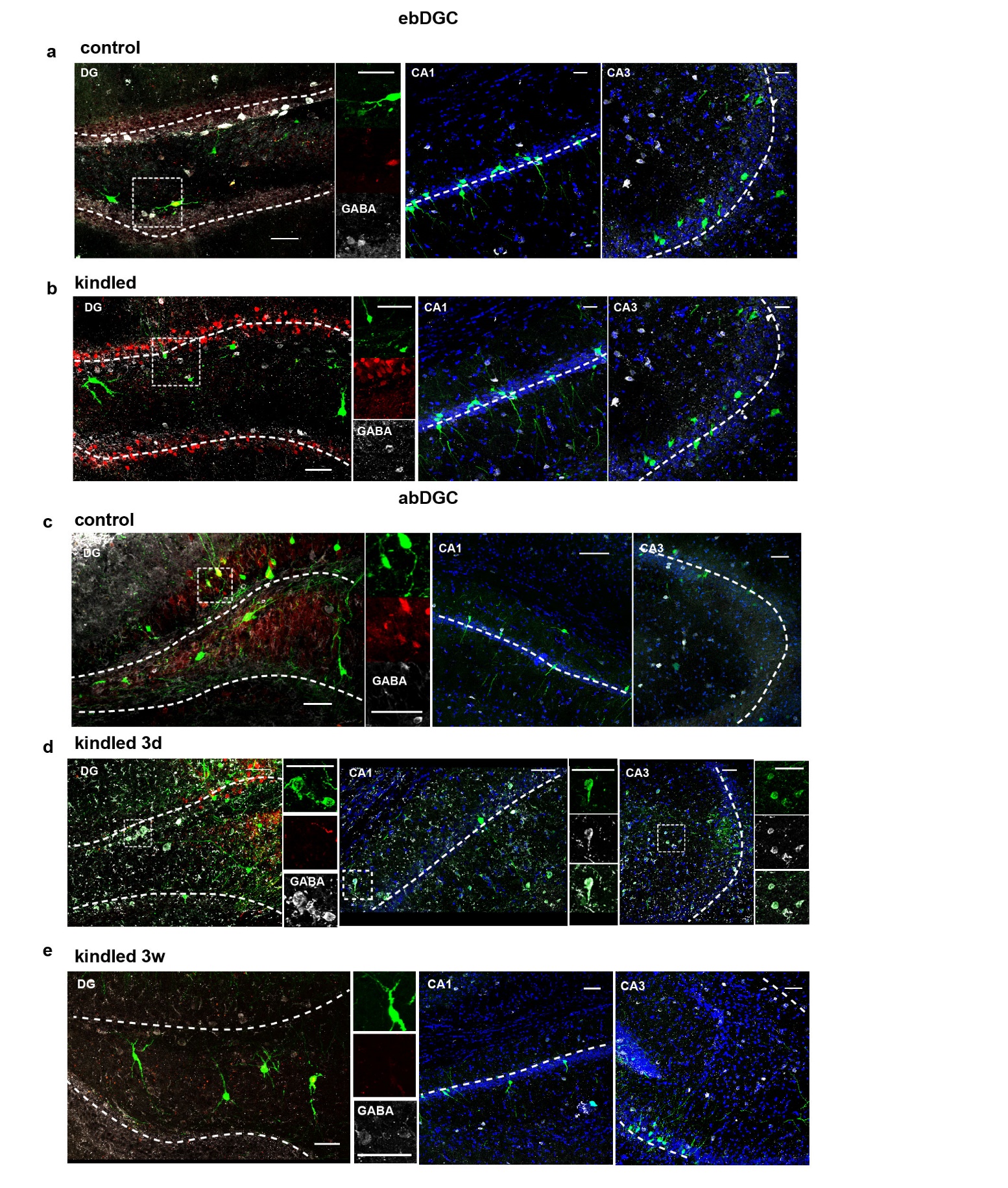


**Figure. S9.**

**GABAergic inputs identified in the DG of ebDGCs and abDGCs born at acute phase and chronic after kindling-induced seizures.** (**a,b**) Double immunostaining of GABA (white) in brain slices with rabies-labeled GFP-expressing presynaptic cells in the DG (left), as well as in the CA1 (middle) and CA3 (right) in non-kindled mice (**a**) and kindled mice (**b**) injected with CamKⅡ-cre and TVA-mCherry viruses (bar=50 μm). (**c-e**) Double immunostaining of GABA (white) in brain slices with rabies-labeled GFP-expressing presynaptic cells in DG, as well as in CA1 and CA3 in non-kindled (**c**), 3 days after fully kindled (**d**), 3 weeks after fully kindled mice (**e**) injected with pUX-cre and TVA-mCherry viruses (bar=50 μm). For DG, the enlarged images show seperately mCherry-, GFP- and GABA- expressing of the selected areas. Only the enlarged images of CA1 and CA3 of mice that are injected viruses 3 days after fully kindled are shown, manifesting the GFP-expressing presynaptic cells colocalized with GABA; whereas, there are few GFP-expressing presynaptic cells colocalized with GABA in CA1 and CA3 observed in brain slices of non-kindled and 3 weeks after fully kindled mice injected with pUX-cre and TVA-mCherry viruses.


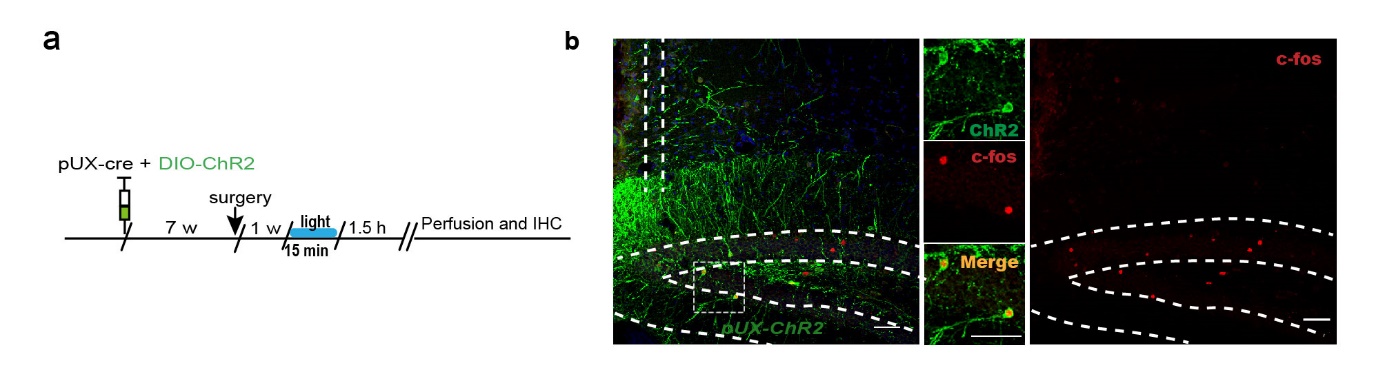


**Figure. S10.**

**Optogenetic activation of abDGCs in naïve mice leads to sparse c-fos expression in GCL.** (**a**) Experimental scheme for optogenetic activation of 8-weeks-old abDGCs in naïve mice. (**b**) Optogenetic activation of abDGCs leads to sparse c-fos expression in the GCL. Immunostaining of c-fos (red) and ChR2-EYFP (green) and the enlarged images (bar=50 μm).


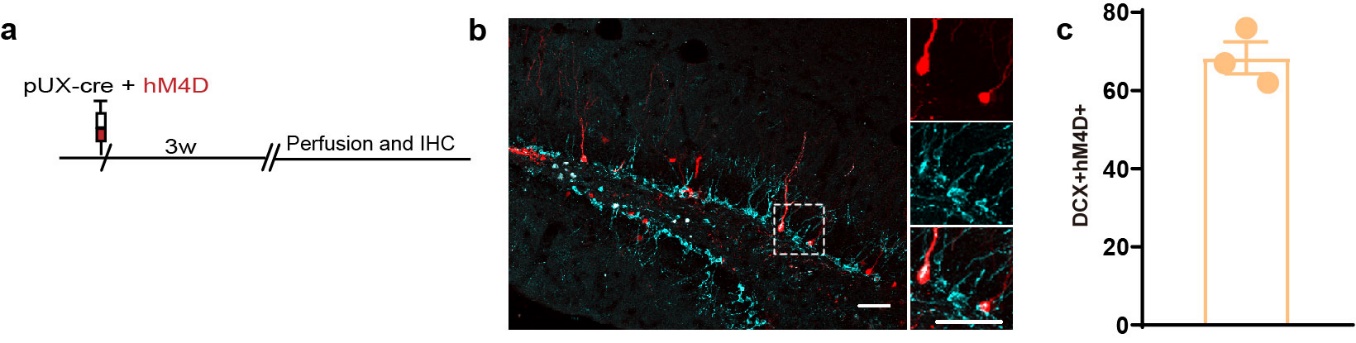


**Figure. S11.**

**Histochemical verification of hM4D-expressing abDGCs labelled with retrovirus.** (**a**) Experiment scheme (mice were perfused 3W post retrovirus injection). (**b**) Representative images of double immunostaining of hM4D (mCherry) with DCX (indigo). Mice were perfused 3W post retrovirus injection (bar = 50 μm). (**c**) Percentage of retrovirus labeled, immunochemically identified (DCX^+^) abDGCs (DCX^++^% = (DCX^+^ hM4D ^+^) / hM4D ^+^×100%; n=3, hM4D^+^ cells were from 3 non-kindled mice, 68.33% were DCX^+^).

Data S1. (separate file)

Source data of all figures and supplementary figures.
